# Microcystin-LR in drinking water: An emerging role of mitochondrial-induced epigenetic modifications and possible mitigation strategies

**DOI:** 10.1016/j.toxrep.2024.101745

**Published:** 2024-09-28

**Authors:** Kashish Gupta, Nikita Soni, Ram Kumar Nema, Neelam Sahu, Rupesh K. Srivastava, Pooja Ratre, Pradyumna Kumar Mishra

**Affiliations:** a Division of Environmental Biotechnology, Genetics & Molecular Biology (EBGMB), ICMR-National Institute for Research in Environmental Health (NIREH), Bhopal, India; b Department of Biotechnology, All India Institute of Medical Sciences (AIIMS), New Delhi, India; c Faculty of Medical Research, Academy of Scientific and Innovative Research (AcSIR), Ghaziabad, India

**Keywords:** Cyanotoxin, Water Pollution, Emerging Pollutant, Mitochondria, Environmental Health

## Abstract

Algal blooms are a serious menace to freshwater bodies all over the world. These blooms typically comprise cyanobacterial outgrowths that produce a heptapeptide toxin, Microcystin-LR (MC-LR). Chronic MC-LR exposure impairs mitochondrial-nuclear crosstalk, ROS generation, activation of DNA damage repair pathways, apoptosis, and calcium homeostasis by interfering with PC/MAPK/RTK/PI3K signaling. The discovery of the toxin's biosynthesis pathways paved the way for the development of molecular techniques for the early detection of microcystin. Phosphatase inhibition-based bioassays, high-performance liquid chromatography, and enzyme-linked immunosorbent tests have recently been employed to identify MC-LR in aquatic ecosystems. Biosensors are an exciting alternative for effective on-site analysis and field-based characterization. Here, we present a synthesis of evidence supporting MC-LR as a mitotoxicant, examine various detection methods, and propose a novel theory for the relevance of MC-LR-induced breakdown of mitochondrial machinery and its myriad biological ramifications in human health and disease.

## Highlights

• MC-LR is one of the most abundant cyanotoxins.
• MC-LR impacts mitochondrial dynamics and interferes with PKC/MAPK/RTK signaling.
• Pathophysiological repercussions are triggered by mitochondrial machinery dysfunction.
• This also leads to a cascade of mitochondrial-epigenetic modifications.
• The article offers novel perspectives on MC-LR as an emerging mitotoxicant.

## Graphical Abstract

**Figure:**
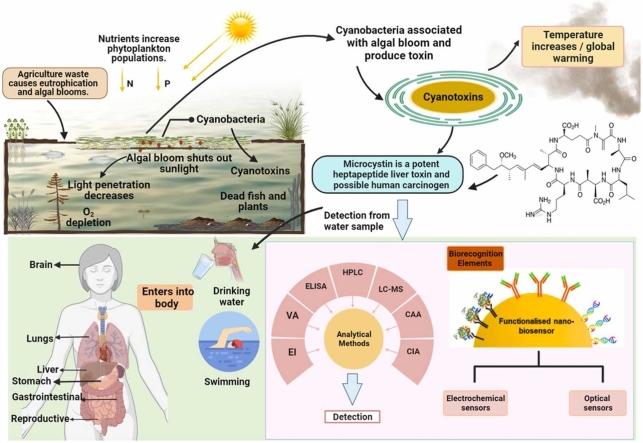

## 1 Introduction

Water is essential to all life for survival. Seawater makes up over 98 % of all water and is hazardous to human health, having an average salinity of about 3.5 %. The remaining 2 % of the world's water supply is freshwater, of which 0.36 % is found underground in aquifers and wells, and 1.6 % is locked in glaciers and polar ice caps. Drinkable water is becoming increasingly scarce because of several anthropogenic activities, both directly and indirectly. These include sewage, metals, nanomaterials (NMs), personal care products (PPCPs), pharmaceuticals, and persistent organic pollutants (POPs), which discharge into water, contaminate, create a stressful environment, and lead to water pollution [1]. Water pollution causes several health problems, including skin ailments (primarily melanosis and keratosis), skin, kidney, liver, and bladder cancer, and water-borne infections, including diarrhea, typhoid, and cholera. Water pollution has become a worldwide issue, and challenges regarding water quality are currently at the forefront of global action. Almost 2 billion people use polluted water sources globally, and it is estimated that nearly 1.5 billion individuals are inaccessible to clean drinking water, posing a great threat to their safety. There are 2.4 billion people in India without adequate sanitation. 90 % of untreated sewage is thrown into bodies of waterbodies, resulting in the annual death toll from illnesses carried by tainted water of almost 5 million people, hurting the ecosystem. The amount of clean water utilized worldwide has increased six-fold over the previous 100 years, according to UNESCO's 2021 Global Water Development Report. World Health Organization (WHO) found that the negative impacts of water pollution on human health account for 80 % of illnesses and 50 % of infant deaths worldwide. Water pollution is mostly brought on by agricultural waste, particularly pesticides, which have a high nutrient load and are coupled with water to generate eutrophication, which results in algal blooms. Algal blooms are expanding rapidly due to rising temperatures and more pollutants. These green waves are an indication of changing climate as well as a warning message. Cyanobacteria significantly aid algal bloom development. As a result of their build-up and breakdown, poisonous secondary metabolites such as hydrogen sulfide, poisons, and odorants are produced. Algal toxins may be released by degrading cyanobacteria, which can be dangerous to aquatic and terrestrial life [2]. Microcystin (MC) is an example of an algal toxin known as cyanotoxin. In addition to Microcystis spp., which is responsible for most MCs, other cyanobacterial species such as *Anabaena, Anabenopsis, Aphanizomenon, Nostoc,* and *Planktothrix* also produce MCs. Microcystin-LR (MC-LR) has leucine and arginine amino acids in the second and fourth positions and is one of the most prevalent kinds of MC [3]. High levels of MCs produced in cyanobacterial blooms, have been reported in various parts of the world. This is due to favorable environmental conditions and the characteristics of the water bodies involved. For instance, levels of 28 µg MC-LR/mL in New Zealand, 23.7 µg MCs/mL in South Africa, 13.6 µg MCs/mL in Uruguay, and 26.1 µg MCs/mL in Uruguay have been recorded [4]. The relatively low probability of adverse health effects is 20,000 cyanobacterial cells/mL, and a moderate probability of adverse health effects is 100,000 cyanobacterial cells/mL [5]. It is a widely recognized algal toxin as it causes toxic effects on mitochondrial machinery, prompting DNA double-strand breaks, producing reactive oxygen species (ROS), impairment of DNA repair, and elevated inflammatory cytokine response leading to various diseases, including cancer [6]. Here, we present a thorough assessment of the evidence supporting MC-LR as a mitotoxicant, explain MC detection techniques, and propose a novel theory for the relevance of MC-induced breakdown of mitochondrial machinery and its myriad biological repercussions in health and disease.

## 2 Cyanobacteria

Cyanobacteria are considered the largest and most diverse class of gram-negative, flagellated, oxygenic, and prokaryotic photoautotrophs, found on rocks, soil, sea, and freshwater as single cells or as colonies [7]. One aspect of the global issue evaluated by the GLOBALHAB Status Report is the occurrence of harmful algal blooms (HABs) in the United States. In East Asia, HABs have been found on the shores of Japan, China, eastern Russia, and Korea [8]. HAB cases have also been found in the Norwegian portion of the Barents Sea, the Norwegian Sea, the eastern North Sea, Kattegat, Skagerrak, and the Baltic Sea [9]. Cyanobacterial blooms occurred in every state in the US, posing a serious threat to the health of humans and animals by producing neurotoxins like anatoxin-A (ATX-a), and saxitoxin (STX) and hepatotoxins like microcystin, nodularin, and cylindrospermopsin (CYN) [10]. Most cases of cyanotoxin poisoning have been seen in domestic animals that consume waters contaminated with cyanobacterial blooms [11]. Thousands of livestock fatalities and numerous poisonings in dogs worldwide have been linked to the ingestion of cyanobacteria [12]. In California, domestic animal poisonings have been linked to blooms of Microcystis sp [13]. Unfortunately, some animals seem to be drawn to cyanobacteria in water and dried crusts of algae on top of the water. Livestock and dogs have been observed drinking infested water even when clean water is available and consuming crust and mats avidly. Blooms of cyanobacterial species that produce microcystins and/or anatoxin-a have coincided with the deaths of ducks, gulls, songbirds, pheasants, hawks, and several other bird species [14]. It has been found that cyanobacteria abundance and the emergence of cyanoHABs resulted in eutrophication in the lakes of the Azores Islands. Several species, including *Aphanizomenon gracile, Microcystis aeruginosa,* and *Raphidiopsis curvata*, were found in the Azores Lake and produced MC, STX, ATX-a, and CYN [15]. Records have been found on the oceanic and continental islands of the Atlantic Ocean between the latitudes of 23°S and 64°N and the longitudes of 13° and 84°W. These islands have a variety of geological settings and climatic conditions, which leads to a wide range of habitat types. 445 taxa of cyanobacteria from 129 genera and 46 groups have been found on islands in the Atlantic Ocean. Brazilian coastal islands (45°-48°W and 23°-25°S), Iceland, Canary Islands, Caribbean islands, Lee Stocking and Cayman Islands (Bahamas), and Curaçau island were found to have cyanobacteria in the Atlantic Ocean [16]. HABs produced by cyanobacteria are poisonous and can cause water discolorations, primarily affecting the Baltic and Brazilian coasts [17]. Individual cyanobacterial cells use exopolysaccharides to link together and create buoyant air-filled intercellular gaps, helping the growth of cyanobacterial blooms [18]. The ability of cyanobacteria to create bioactive secondary metabolites is a common way to classify them as toxic or non-toxic, depending on the temperature of the water [19]. 70 % of global freshwater blooms are estimated to be toxic. In 1996, 50 dialysis patients died in Caruaru, Brazil, after drinking water contaminated with MCs. In May 2007, over two million individuals in the city of Wuxi were affected by a drinking water crisis brought on by a cyanobacteria bloom in Taihu Lake (Wuxi, China). Over 500,000 individuals received a warning not to drink water in August 2014 when it was discovered that the MC concentration in the water was higher than the legal limit of 1 g L^−1^. Cyanobacteria blooms are spreading, posing a threat to the viability of aquatic habitats in areas like Lake Erie in the United States, Taihu Lake (China), Lake Nieuwe Meer (Netherlands), and Lake Winnipeg (Canada). WHO recommends alert levels for cyanobacteria based on chlorophyll concentrations: 1 µg/L and 12 µg/L for Alert Levels 1 and 2 respectively. The WHO alert level framework recommends utilities to enhance their monitoring activities for cyanotoxins if cyanobacterial biovolume exceeds 0.3 mm^3^/L (Alert Level 1). In addition, utilities should explore methods to limit the entry of cells into the plant or implement steps to address possible cyanotoxins. Over time, these toxins may reach the recommended levels for microcystins, anatoxin-a, or saxitoxin. Alert Level 2 is triggered when the biovolume surpasses 4 mm^3^/L, indicating a possible acute toxin hazard according to the short-term recommended levels for cyanotoxins in drinking water [20]. The alert level framework is less applicable to cylindrospermopsin as it may be actively released by cyanobacteria cells, so cyanobacterial biomass is a poor indicator of this toxin [21]. The CyanoFluor displayed high sensitivity (limit of quantification = 3.5 µg L^−1^ of phycocyanin) and could detect cyanobacterial biovolumes to levels much lower than the threshold levels in current recreational guidelines worldwide [22]. WHO Alert Level 1 for cyanobacterial blooms in recreational waters occurs when the chlorophyll-a level exceeds 12 micrograms per litre and cyanobacteria dominate. At this level, the potential health risk to humans is considered moderate [23]. Due to the extensive use of freshwater systems, the nutrient load increases, leading to frequent cyanobacterial blooms. These cyanobacterial toxins have frequently been observed in many Chinese lakes, such as Lakes Taihu, Chaohu, Dianchi, Sri Lanka, India, Singapore, and other Asian countries [24]. WHO recommended a provisional 1 µg/L MC-LR guidelines for drinking water quality. The levels of MC-LR in the lakes and dugout ponds of Alberta, Canada, ranged from 4 to 605 µg/g dry weights of biomass [25] or up to 1500 µg/g. Between 1990 and 1992, more than 70 % of over 380 bloom biomass samples from 19 lakes in Alberta showed detectable levels (>1 µg of MC-LR per g dry weight of biomass) of toxin [26]. Similarly, levels of MC-LR from natural blooms of Microcystis in Japan between 1989 and 1991 ranged from 27 to 622 µg/g dry weight of biomass. In the same blooms, the levels of MC-RR and MC-YR ranged from 11 to 979 µg/g dry weight of biomass and from 9 to 356 µg/g dry weight of biomass, respectively, with a total maximum level of microcystins of 1732 µg/g dry weight of biomass [27].

When cyanobacteria blooms occur, many dead cyanobacteria settle to the bottom and decay. As they do so, they consume oxygen, lowering the water's dissolved oxygen (DO) level. These blooms acquire all the available oxygen for their multiplication, due to which there is significantly less or no oxygen available for other aquatic flora (*Lepidium sativum, Oryzasativa L*.), fauna (fish, shellfish, and invertebrates), leading to their extinction, as well as a reduction in the species diversity of the marine ecosystem and also affect the human health [28]. When cyanobacteria undergo rapid metabolism and multiplication or lysis and senescence, cyanotoxins can be secreted actively. They have various structural characteristics and modes of action [29]. From a human perspective, cyanobacteria have both advantageous and harmful characteristics. Exposure to cyanobacteria-blooming water changed the kidneys' mRNA and protein translation patterns of ER stress signaling components [30]. These cyanobacteria-related water quality issues can significantly impact human health and the economy, and they have attracted research interest and raised public concern [31].

### 2.1 Green algal blooms

Microalgae play a crucial role as primary producers and contribute significantly to energy flow, ecological equilibrium, nitrogen fixation, material cycling, and pollution breakdown. Additionally, they accumulate contaminants or pollutants, which can lead to the direct degradation of the ecosystem[32]. While many algae offer benefits, microalgae sometimes trigger the formation of algal blooms (ABs), particularly in aquatic environments, both saltwater favorable and freshwater, when they become densely packed or experience rapid growth due to promising conditions. They can lead to anoxia and high biomass accumulation [33]. They excessively proliferate in a water body, resulting in toxicity caused by either the toxins themselves or the accumulation of biomass, which disrupts the coexistence of organisms in the environment [34]. These blooms may be identified by three distinct features: the poisoning of freshwater and marine organisms, the lack of oxygen caused by a large amount of biomass, and the buildup of poisons in the food chain [35]. Cyanobacteria genera commonly associated with bloom formation include *Dolichospermum*, *Microcystis, Aphanizomenon, Trichodesmium, Nodularia, Cylindrospermopsis,* and *Planktothrix*. Studies conducted in various freshwater ecosystems across North America, Asia, Oceania, Africa, and Europe have focused on the potential impact of cyanobacterial harmful algal blooms (cHABs) on human health [36]. These global research findings highlight the critical need for all-encompassing approaches to reduce the hazards of cHABs and protect the environment and human health.

### 2.2 Cyanotoxins

Cyanobacteria release unique compounds that aid their survival and preserve their dwellings under adverse environmental conditions. These are low molecular weight metabolites with unique bioactive qualities of several chemical groups, including polyketides, carbohydrates, terpenes, peptides, phenolics, and alkaloids [37]. This exceptional characteristic causes cyanobacteria to form toxic blooms, cyanotoxin, which could risk human health. Cyanobacteria are characterized by gene sequences that code for producing toxic metabolites; various genera of cyanobacteria release these toxins. These genera include *Cylindrospermopsis, Dolichospermum* (previously Anabaena), *Microcystis*, and *Planktothrix*, which dominate the cyanoHAB (harmful cyanobacterial bloom) community [38]. These toxins are lethal to humans and include neurotoxins (antitoxins and saxitoxins), hepatotoxins (MCs and nodularin), cytotoxins (CYN), cutaneous toxins (Aplysia toxin and Lyngby toxin), dermato-toxins, irritating toxins (lipopolysaccharides) and endotoxins [39]. The MCs are produced through a complex protein assembly that accommodates non-ribosomal peptide synthetase (NRPS) and polyketide synthase (PKS) domains [40]. This sophisticated protein complex originates from extensive bacterial gene clusters, consisting of at least two operons: the mcyABC (peptide synthetase) and mcyDE (hybrid polyketide-peptide synthetase) [41]. Their key feature is that they are non-strain specific, which means that different cyanobacteria genera can synthesize the same cyanotoxin and vice-versa [42]. Cyanotoxins regarded as one of the deadliest classes of biotoxins yet discovered, with significant hazards for disease and mortality [43]. The generation of cyanotoxins in fishable, potable, and recreational water resources is the primary health risk brought on by cyanoHABs. Consuming fish or other foods from contaminated water can expose humans to cyanotoxins, which harm health. Cyanotoxins are produced by cyanobacteria found in lakes and oceans, and they can rapidly reproduce in high phosphorus concentrations. When cyanobacteria bloom, they can produce cyanotoxins in concentrations that poison or kill animals and humans [44]. Numerous traditional water treatment methods, including coagulation, flocculation, sedimentation, and filtration, struggle to efficiently eliminate extracellular/dissolved cyanotoxins due to their fluctuating concentrations and types [45]. Some of them, including MCs and CYNs, have been found to cause DNA deterioration (genotoxicity) and promote carcinogenicity [46]. Cyanotoxins were discovered in 131 freshwater ecosystems in countries, primarily in lakes, ponds, and reservoirs, in 273 freshwater habitats in Europe across 25 nations. In 204 freshwater environments, mostly lakes, cyanotoxins were discovered in 4 North and Central American countries. Cyanotoxin intoxication was documented in Australia when 148 persons, predominantly children, were hospitalized with gastrointestinal symptoms due to drinking water polluted with the cytotoxin CYNs [47]. Several neurotoxin cases were reported in France, where animals died after drinking water contaminated with anatoxins. Cyanotoxins were discovered in 79 freshwater ecosystems throughout six nations in South America, in reservoirs and lakes. Cyanotoxins, such as ATXs (3 %), CYNs (10 %), STXs (1 %), and NODs (3 %), have also been found in Asia. In 14 countries and 76 freshwater environments, MCs (77 %; 77 out of 100) were the most prominent cyanotoxins reported in Africa's lakes, reservoirs, and ponds [48]. Neurotoxins are the most harmful cyanotoxins, and MCs and CYNs are the two most investigated worldwide. Most cyanotoxins in freshwater ecosystems in Asia were MCs (132 out of 168) at 79 % [49].

### 2.3 Types of cyanotoxins

#### 2.3.1 Cyclindrospermopsin

The compound cylindrospermopsin has a molecular weight of 415 Da and functions as a zwitterion. It consists of a tricyclic guanidine end attached to hydroxymethyl uracil [50]. The toxins CYN comes in two different forms, 7-epi-CYN, and 7-deoxy-CYN. It was initially found in blue-green algae named *Raphidiopsis raciborskii* in 1992. *Aphanizomenon, Lyngbya, Rhadiopsis, Planktothrix*, and many other filamentous freshwater cyanobacteria of the order *Oscillatorials* and *Nostocales* were later shown to produce CYNs. Although anaerobic conditions can prevent CYN deterioration, it is a stable substance in various environmental conditions like UV and visible light, adaptable pH, and temperature. When people consume CYN-infected food or drinks (such as fish, shellfish, vegetables, or algal supplements) or accidentally consume contaminated water while participating in recreational activities like swimming or boating. In 1997, Queensland experienced the first livestock death attributed to CYN. CYN has primarily been detected in Thailand, Taiwan, Japan, Turkey, China, and Saudi Arabia [51].

#### 2.3.2 Nodularins (NODs)

It is a cyclic pentapeptide. L-arginine, N-methyldehydrobutyrine, D-glutamic acid, and D-erythro-methylaspartic acid make up the structure of NODs. The most typical cyanotoxin among the ten analogs is NOD-R. NODs were first discovered in cyanobacteria's freshwater and coastal environments, known as Nodularia [52]. The newly discovered NODs were obtained from the Australian cyanobacterium *Iningainema pulvinus* (Scytonemataceae). They are anticipated to have toxic mechanisms and structures closely associated with MC, hence similar detection procedures. It is found in Taiwan and Türkiye.

#### 2.3.3 Saxitoxin

Paralytic shellfish toxin (PST) is another name for it. Its neurotoxic properties are frequently linked to red tides because of marine dinoflagellates such as (*Gymnodinium sp., Pyrodinium sp.,* and *Alexandrium sp*). This toxin is produced by various taxa of freshwater cyanobacteria, including *Lyngbya, Anabaena*, and *Aphanizomenon*. Saxitoxins, also known as goniototoxins and Ctoxins, are a class of unsulfonated, monosulfonated, or disulfonated carbamate alkaloid neurotoxins. It is made up of 3, 4, and 2 guanidine groups–containing a tricyclic system. As a neurotoxin, it blocks open, reversible, voltage-gated sodium channels, preventing sodium ions from passing through the membrane and resulting in the nervous system's shutdown. Saxitoxin was detected once in China and Russia [50].

#### 2.3.4 Anatoxin-a

It is the smallest cyanotoxin, having a molecular mass of 165 Da and a bicyclic secondary amine structure with neurotoxic properties [53]. It is also called Very Fast Death Factor (VFDF) secondary metabolites. This toxin is produced globally by cyanobacterial species belonging to the *Planktothrix, Anabaena,* and *Aphanizomenon* genera. Anatoxin-A toxicity can be fatal owing to respiratory paralysis and includes loss of coordination, muscle fasciculation, and convulsions. Its mechanism of action is the nicotinic acetylcholine receptor (nAchR), which mimics the binding of its natural ligand, acetylcholine. Considering its high toxicity and occurrence in drinking water, it presents a significant concern to animals and people [54].

#### 2.3.5 Microcystin

Among all, the most common and harmful type of cyanobacterial toxins is MCs, often found in freshwater, estuaries, and coastal environments. Some cyanobacteria genera, including *Microcystis, Anabaena, Nostoc, Limnothrix, Phormidium, Cichlidium, Oscillatoria*, etc., produce MCs [55]. The first cyanobacterial species to be identified as producing MC-LR is *Microcystis aeruginosa* [56]. It is a hepatotoxin; the liver is the main organ MC-LR targets. The liver-specific organic anion-transporting polypeptide membrane transport mechanism has led to the liver being recognized as the organ that MC-LR specifically targets [57]. Growing data suggest that MC-LR can cause hepatocytes to induce apoptosis and necrosis, influence how these cells regulate their immune systems, and even encourage hepatocellular cancer. MC-LR exposure results in cellular damage, hepatotoxicity, and genotoxicity. MC-LR also decreased the expression of the HNF4A gene, a critical regulator of hepatocyte differentiation and function. Genes encoding hepatobiliary membrane transporters (OATP1B1, BSEP, NTCP) [58]. MC-LR has been extensively studied because of its toxicity in vertebrates, such as humans, mice, and fish. MC toxicity is caused by cellular absorption, interactions with protein phosphatases, oxidative stress development, and triggering apoptosis. It has also been shown that MC-LR can lead to neurotoxicity and malfunctioning of the lungs, kidneys, and pancreatic islets [59]. Exposure concentration of MC-LR was significantly correlated with genotoxicity and neurotoxicity levels in both fish and mammals (p < 0.05) [60]. The presence of MC-LR resulted in a notable decrease in the expression of structural components of the blood-brain barrier (CLDN1). In contrast, it led to a significant increase in the expression of genes associated with inflammation (NLRP3, TNF, IL-1β, and CXCL12). Furthermore, the expression of genes associated with neuronal development (LGALS1, CACNA2D2, and NLGN4X) and neurotransmitter transmission (SLC6A13 and AChE) was notably decreased [61]. Chronic exposure to low MC levels is among human health's most significant elements [62]. Previous studies revealed MC-LR stress, suggesting that impairment of the peroxisome proliferator-activated receptor signaling pathway upregulated fatty acid biosynthesis and elongation to promote lipid accumulation [63]. Due to this, diarrhea, neuro-paralysis, liver damage, toxicity, and even death may appear in extreme circumstances. These symptoms could be brought on by epigenetic modification or variations in the mitochondrial DNA sequence. By altering the balance of ROS, MC-LR suppresses immunity and causes apoptosis in vertebrates [64].

## 3 MC-LR exposure and potential toxicity

Humans can be exposed to MC-LR from recreational activities, drinking water, fish, shellfish, vegetables, and dietary supplements made from algae [65]. Following the oral route, MC-LR is absorbed (7–10 %) in the gastrointestinal tract and is diffused into the bloodstream. Once MC-LR is circulated, it quickly moves to blood-irrigated organs like the lungs, kidneys, brain, and liver [66] and enters the cell from the bloodstream, causing mutation, changes in the cell's morphology, cytoskeletal dysfunction, mitochondrial alteration, etc. MCs trigger toxicity by a variety of cellular and molecular mechanisms. These big hydrophilic molecules are expected to pass into cells through transmembrane organic anion transporter peptides (OATP), which control the sodium-independent solute passage [67].

### 3.1 Cellular and molecular mechanisms

MCs cannot diffuse through plasma membranes because of their high molecular weight and complex structure. Numerous pathways also absorb MCs due to their organotropism and cell selectivity. After internalizing the cell, with the help of OATP1B1 and OATP1B3, it starts striking cytotoxic effects on cytoskeleton disruption [68]. Hepatocytes, enterocytes, and vital organs, including the lung, heart, spleen, brain, pancreas, and BBB, all involve OATP multi-specific transporters. The blood perfusion rate, the types of OATP transporters, and their expression levels will all impact how the MC is distributed throughout the cells of the body and organs. High expression of OATPs in the liver, most consumed MC accumulates in hepatocytes. Most of the percentage of MCLR is excreted out by the biliary route, approximately 15 % of ingested MC-LR is excreted in the faces, and the remaining percentage of MC-LR accumulates in the cells. PP1 and PP2A bind to MC-LR in two steps, resulting in sub-cellular toxicity. Toxin first inactivates the enzyme by binding to it, and then, throughout an extended reaction time, the covalent bond forms. The dynamic process of protein phosphorylation and dephosphorylation is a vital way of regulating the activity of proteins in cells. One of the most studied MC activity mechanisms is the suppression of serine/threonine protein phosphatases (PP1/PP2A) by interacting via their catalytic components [69]. The initial suppression of PP2A via MC treatment and the hyperphosphorylation of proteins like p53 results in various events that lead to cellular death through apoptosis or necrosis [70]. The interaction of microcystins with cellular transport mechanisms profoundly impacts multiple organ systems, especially the liver. Microcystins contribute to severe cytotoxic effects and cell death by disrupting regulatory pathways and promoting cellular stress. Understanding these processes is crucial for developing interventions to mitigate health risks associated with chronic exposure to microcystins.

Cellular processes are adversely affected by a series of events brought on by MCs' hyperphosphorylation of PP2A. The uncontrolled suppression of these enzymes may significantly impact the homeostasis of the cell. The MC-LR interacts with hydrophobic grooves, C-terminal grooves, and the catalytic domain of the enzyme to bind to the PP-1c subtype. To stop molecules from entering the active center of the enzyme, MC-LR’s MeAsp residue binds with the Tyr134 and Arg96 of PP-1c. Interactions may also happen at the PP-1c's toxin-sensitive b12-b13 loop. Furthermore, the activity of PP1/PP2A and an elevation in the phosphorylation of cytoskeleton proteins have been correlated to the widely recognized effects of MC-LR on the hepatic system and structure of the cytoskeleton [71]. Additionally, the direct inhibition of PP2A causes phosphorylation that decreases the protein kinase function of DNA-PK, which in turn causes inhibition of the DSB-NHEJ repair pathways. CaMKII activation by caspases may also control downstream events, including ROS generation and protein phosphorylation like myosin light chain (MLC). A rise in intracellular Ca^2+^ activates the enzyme and participates in the later stages of MC-induced apoptosis [72]. The over-phosphorylation of PP2A by MC-LR disrupts cellular processes, leading to changes in cell homeostasis. This, along with CaMKII activation and increased intracellular Ca^2+^, contributes to MC-LR's impact on cellular apoptosis and liver function. Understanding these mechanisms is crucial for developing strategies to mitigate MC-LR's toxic effects and protect cellular health.

The effect of severe stress produced in the mitochondrial milieu on retrograde signaling and Nrf2 levels, a crucial mediator of mitochondrial-nuclear cross-talk, starts a chain reaction of atypical epigenetic modifications, such as altered histone tails, hypomethylated DNA, and altered expression of mitochondrial miRNAs [73]. Previous research on one of the most hazardous isocyanates is methyl isocyanate (MIC), a combative industrialized waste that is believed to have negative outcomes on many organ systems and immunity, triggering apoptosis by DNA fragmentation, externalization of PS and depolarization of the mitochondrial membrane potential and induction of mitochondrial-mediated events. By regulating the restoration of damaged DNA and cell death in reaction to stress, the phosphoinositide 3-kinase-like kinase (PIKK) ataxia telangiectasia mutated (ATM) kinase is crucial for maintaining genome fidelity. When ATM is autophosphorylated at Serine 1981 residue, the following signaling molecules in the cascade are activated, thereby repairing the damage. In response to DNA damage, the histone H2A protein family's alternative isoform H2AX undergoes phosphorylation on serine 139 residue, resulting in the creation of massive, discrete nuclear H2AX foci that allow the packing of damaged DNA into apoptotic bodies [74].

**Fig. 1:**
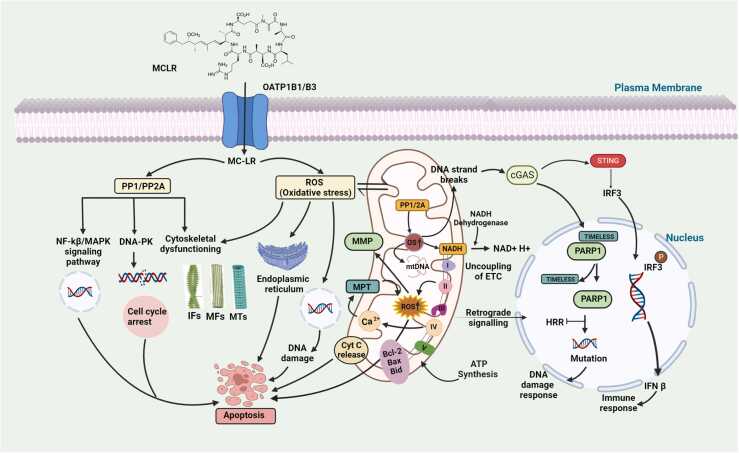
Schematic representation of the molecular mechanisms driving the toxicity of microcystins (MC). After being taken in by the OATP through the plasma membrane, the serine/threonine protein phosphatases (PP1/PP2A), to which MC binds specifically, are inhibited, which sets off a series of events that cause cytotoxicity and genotoxicity in animal cells. Abbreviations: ATP: Adenosine triphosphate; Bax: Bcl-2-associated X protein; Bcl-2: B-cell leukemia/lymphoma 2 protein; Bid: BH3 Interacting-domain death agonist; cGAS: Cyclic GMP-AMP synthase; Cyt C: Cytochrome complex; DNA: Deoxyribonucleic acid; DNA-PK: DNA-dependent protein kinase; ETC: Electron transport chain; HRR: Homologous recombination repair; IFN β: Interferon beta-1; IFS: Intermediate filaments; IRF3: Interferon regulatory factor 3; MCLR: Microcystin-Leucine arginine; MC-LR: Microcystin-Leucine Arginine; MFS: Microfilaments; MMP: Mitochondrial membrane potential; MPT: Membrane-permeabilization transition; MTs: Microtubules; NAD+: Nicotinamide adenine dinucleotide; NADH: Nicotinamide adenine dinucleotide; NF-kβ: Nuclear factor kappa-light-chain-enhancer of activated B cells; OATP: Organic anion transporter peptides; OATP: Organic anion transporter peptides; OS: Oxidative stress; PARP1: Poly (ADP-ribose) polymerase 1; PP1: Protein phosphatase-1; PP2A: Protein phosphatase 2A; ROS: Reactive oxygen species; STING: Stimulator of interferon genes.

ROS are inextricably connected to mitochondrial metabolism and may lead to cell apoptosis, necrosis, or genotoxicity. Ca^2+^ is, therefore, the primary mechanism causing MC-LR-triggered MPT and cell death [75]. Elevated ROS can cause cytoskeletal alterations and generate oxidative DNA damage, which could cause cancer growth and apoptosis by three major pathways including the increase of ROS formation, disturbance of mitochondrial membrane potential (MMP) and release of cytochrome c (cyt c) from membrane-permeabilization transition (MPT) and shows cytotoxicity at the early stages of apoptosis (Table 1) [76], [77]. Furthermore, it has been demonstrated that oxidative stress could cause c-Jun N-terminal kinases (JNKs) pathway to activate, leading to mitochondrial dysfunction. Nek2, a cycle-reliant protein kinase, regulates mitotic progression and chromosomal segregation. The PP1 holoenzyme and Nek2 kinase make a complex. The complex is bound by MC-LR, which activates Nek2 kinase. This biochemical interaction may occur at the cellular level, potentially affecting cell survival, tissue damage, and tumor growth [71]. Additionally, gross chromosomal rearrangements (GCRs) are caused by DNA damage from oxygen metabolism and ROS. The cyclin-dependent kinase inhibitor p21 (Cip1/WAF1/Sdi1) and the cancer suppressor p53, which restricts proliferation by preventing the entrance into the S phase, are expressed by cells in response to oxygen-induced damage. p53 is significantly impacted by cellular insults that change mitochondrial function, and ROS produced at mitochondria are believed to control p53 activity [74]. Changes in cyclin-dependent kinase (CDK) activity frequently underlie tumor-associated cell cycle abnormalities, which cause unpremeditated proliferation and chromosomal instability. In response to cellular stress, the nuclear phosphoprotein p53 is activated. p53 controls both pro- and anti-apoptotic gene expression, including Bcl-2 and Bax, which are Bcl-2 family member. These proteins participate in p53-induced cell death by aiding with mitochondria's outer MPT, which triggers the apoptotic process. Our research suggests that p53 is essential in inhibiting the proliferation response and tumor development brought on by long-term, sublethal MC-LR exposure. p53 may be activated in toxin-exposed cells as a defensive mechanism against cell processes that promote cell growth and tumorigenesis, causing apoptosis. Bcl-2 is another protein that may have a role in MC toxicity. This protein is found in the mitochondria and endoplasmic reticulum and is controlled by PP2A or p53. Excessive phosphorylation of p53 may trigger apoptosis by disrupting mitochondria, phosphorylating mitogen-activated protein kinases MAPKs to modify the cytoskeleton, and phosphorylating CaMKII to produce ROS species. Proto-oncogene expression is controlled by MAPKs by PP2A and is regulated by MC which controls the gene transcription associated with cell development and differentiation (Fig. 1). The MC-LR mediated toxicity is also linked with impaired mitochondrial machinery, which includes mitochondrial mutation and can be caused by abnormalities in nuclear-encoded mitochondrial proteins, mtDNA-coded proteins, or altered apoptotic and survival signaling pathways [78]. The interaction of ROS, mitochondrial dysfunction, and cellular stress responses is crucial for understanding MC-LR-induced toxicity. Elevated ROS levels disrupt mitochondrial function, leading to oxidative DNA damage and apoptosis. Targeting these pathways may help mitigate the harmful effects of MC-LR.

**Table 1:** Table showing different signaling pathways through which MC-LR causes human health effects.

S. No.	Cell-line	Dose of MC-LR	Protein/factor	Signaling pathway involved	Repercussion	Toxicity/Phenotypic effects to be address	References
1	HL−7702	0.01–1 μM	BLK, cGAS	cGAS-STING signaling pathway	Cause an immune-mediated response and mutation	Hepatic disease	[133]
2	Human gastric cancer cell line (SGC−9701)	0–10 μM	Krt16 and ERα	Estrogen signaling pathway	Proliferation, invasion, migration, anti-apoptosis activity	Gastric cancer	[134]
3	Human uroepithelial cells (SV-HUC−1)	1–1000 nM	Cyclin D1	PI3K/AKT/GSK3β/ mTOR/HIF−1α/VEGFCyclin D1 and JAK2/STAT3/Bcl2 signaling pathways	Proliferation of urinary bladder, SV-HUC−1 cell angiogenesis	Bladder cancer	[135]
4	Human aortic vascular smooth muscle cell line (HAoSMC)	0.01–1 μM	Caspase−3 and caspase−9	Integrin β1, Rho, ROCK, MLC, and integrin-mediated FAK/ROCK signaling pathway	ROS generation, MMP loss, cytoskeleton impairment, S-phase arrest, and cell death	Hinder the migration and proliferating of HAVSMCs	[136]
5	RWPE−1 cells (epithelial cell-derived cell line)	10–500 nM	MMP−2, MMP−9	FOXM1/COX2	Triggering of malignant phenotype in human prostate epithelial cells	Prostate Cancer	[104]
6	Human hepatic stellate cell line (LX−2)	500 nM	α-SMA, collagen I, Gli1/2	Hedgehog	Proliferation, fibrosis, prevent cell viability	Hepatic fibrosis	[137]
7	HL−7702	1 μM, 5 μM and 10 μM	IRS1, Akt, GSK−3, GS, PP2A and INS signaling protein	Insulin signaling pathway,	Affect the phosphorylation levels of proteins	Toxic effect hepatic glucose metabolism	[138]
8	HepaRG	10, 100 and 1000 ng mL^−1^	Nrf−2, CHOP, NOXA, JUN and FOS, NF-κβ,	PP1, PP2, UPR, p53, DR5, GADD34, ATF3, MAPk/p38	Morphological changes, raised ROS and oxidative stress, accumulation of in outpace of ER	Damage to liver	[139]
9	Human hepatoma cell line (HepG2 and SMMC−7721)	0–10 µM	PP2A/MAPK/p53, cdc25C, and cdc2	MAPK/PP2A pathway	Migration, invasion and HepG2 proliferation	Hepatocellular carcinoma (HCC)	[76]
10	HepG2	1 µg/mL	PP1A and PP2A	-	Oxidative stress and damage to DNA	Cytotoxicity and genotoxic	[140]
11	HL7702	0–10 µM	FoxO	FoxO, KEGG, PI3K-AKT, MAPK, Ras signaling pathway, cell cycle	Affect the hepatic circRNAs, toxicity to liver, ER stress	Liver damage	[141]
12	Human normal intestinal epithelium cells (NCM460)	5 or 10 μM	p-p53	PP2A	DNA damage, elevated ROS level	Genotoxicity	[142]
13	Human neuroblastoma cell line (SK-N-SH)	15 or 30 μmol/L	p62 and LC3 II/LC3 I	-	Cell viability, accumulation of autophagosomes, increased concentration of free intracellular calcium	Neuronal toxicity	[143]
14	Human normal liver cell line (LO2)	10 μg/L	p53, Bax, CTNNB1 and MMP7	Wnt/β-catenin and p53 signaling pathways	Programmed cell death.	HCC	[144]
15	Human neuroblastoma cell line (SH-SY5Y)	20–100 mg/mL	OATPs, tau protein	-	Reduced cell viability, neurological degeneration, production of oxidative stress and disruption of AChE	Neurotoxic effects	[145]
16	HL7702	5 and 10 µM	AKT1, p-AKT1	-	DNA damage, cell proliferation and migration	Hepatotoxicity	[146]
17	Human colon cancer cell line (DLD−1 cells)	0–50 nM	-	MiR−221/PTEN and STAT3 s	-	Migration of colon cancer	[147]
18	HL7702	0, 5 or 10 µM	VASP, PP2A	MAPK/ERK1/2 pathway	Raised microfilament depolarization, cytoskeletal destruction, tumor formation, proliferation of cells and cellular death	Hepatotoxicity	[148]
19	HL7702	0–50 µM	F-actin α-tubulin, hyperphosphorylated cytoskeletal-related proteins heat shock protein 27, Tau, and VASP	P44/42 MAPK (ERK1/2)	Decreased viability and prevent hepatocyte adherence	Decreases cellular adhesion in a human liver cell line	[149]
20	L02 cells	0–100 nM	Gankyrin expression, c-myc	Akt-MAPK	ROS production, L02 cell proliferation, movement, colony formation	Malignant transformation	[150]
21	HL7702	0–10 µM	-	Akt, GS, GSK3, PP2A and PP1	Inactivate GS levels	Insulin signaling disruption	[151]
22	A549 human non-small cell lung cancer cell line (ATCC-CCL−185)	0–10 µM	PP2A, Ezrin, VASP and HSP27	MAPK, PP2A-centered pathway	Molecular and cellular changes, microtubule, and filamentous actin rearrangements	Cancer	[152]
23	Hep2 cells	0.5–10 µM	PP2A/A, PP2A/C, PP2A/B55a and B56a, vimentin, VASP, p-Tau and Ezrin	PP2A, p38 and ERK1/2, p38 and ERK1/2,	Chane the protein level and post-translational modification of PP2A subunits, cytoskeletal reorganization, retracted cell morphology, migration, and affect the cell cycle, and changes to the cytoskeleton-associated proteins	Migration of tumor cells	[153]
24	DLD−1, human colorectal adenocarcinoma cell line (HT−29), and SW480	0–50 nM	MP−13	PI3-K/AKT	Tendency of colorectal cancer cells to invade was changed, raised MMP−13 Expression	Increased propensity for spreading and invading of colorectal cancer cells	[154]
25	HL−7702	0–10 µM	PP2A/C, Akt, S6K1, mTORC1, p-Akt (both T308 and S473), p-S6K1, p-S6, and p−4E-BP1, p-c-Myc (S62) and p-c-Jun (S63), Bcl−2, Bad, Cdk1	Akt/S6K1, PP2A	Promote cell growth, increase cell resistance to apoptosis, and Increased c-Myc expression promotes carcinogenesis and cell transformation.	Higher tumorigenic risk	[155]
26	Human normal liver cell line (HL7702)	0–10 µM	Bcl−2, Bad, PP2A	c-Myc, c-Jun and Akt/ S6K1 pathway,	Rearrangement of the cytoskeleton and alteration in the cell proliferation rate	Promotes HL7702 cell growth	[155]
27	ACHN (Human renal adenocarcinoma and HEK−293	0.1–200.0 µM	Bax, p53, Survivin	Caspase 3 and caspase 9	Pro-apoptotic impacts, cell survival, and the morphological alterations of apoptosis, cellular damage, increase in the expression of Bax and p53	Cytotoxicity	[156]
28	The human normal liver cell line HL−7702	5–10 µM	Tau, VASP	PP2A, MAPK, p38 MAPK	Increased intranuclear localization, cytoskeletal reorganization	Liver toxicity	[115]
29	HL7702	0.001–10 lM	Ezrin, VASP, cofilin, and ARP2/3	P38, ERK1/2, MAPK, PP2A	Rearrangement and depolymerization of Actin filament	Hepatotoxicity	[157]
30	human hepatocellular carcinoma cell line (SMMC−7721)	0–10 µM	α4, HSP27, VASP, Rac1 activity, PP2A/C, cofilin	PP2A, PKA	Cell proliferation, reorganized cytoskeleton, and hyperphosphorylation of cytoskeleton-associated proteins.	Cytoskeletal reconfiguration	[158]
31	HL−7702	5–10 µM	Tau, VASP	PP2A, MAPK, p38 MAPK	Raised intranuclear localization and reorganized cytoskeleton.	Liver toxicity	[159]
32	Human melanoma cancer Cell (MDA-MB−435)	0–50 nM	MMP−2, MMP−9, NF-κB	PI3-K/AKT	Cell invasion promotes tumor growth, cancer progression, and the onset of apoptosis in melanoma cells.	Promote cancer metastasis	[160]
33	Human hepatoma-derived cell line (Huh7)	0.5–100 µM	p53, PP2Ac, ATF−4, BiP, NF-κB, IFN-α, ATF−4, CHOP, CREB	PP2A, IRE1, PERK,	Antiviral defense, restriction of cell proliferation, and apoptosis encourage tumor growth	Inflammation, tumor-causing toxic effect on liver	[161]
34	HL7702	0.001–10 M	K8/18 and vimentin, p-K18 (ser52)	MAPK, PPA2	Cell death, inhibits cellular growth, IFS reorganization	Hepatotoxicity	[162]
35	WRL−68	10 µg/L	p27Kip1, p57Kip2, BCL2, TNF-α, SREBF1	Akt/proteinkinase B, PI3K	Altered miRNA expression.	Tumorigenesis	[163]
36	HL−7702	5 or 10 µM	HSP27, F-actin, α-tubulin	PP2A, MAPKs, ERK1/2, phosphorylated JNK and phosphorylated p38 MAPK	Microtubule and actin filament reorganization, cytoskeleton modifications, and minor changes in cellular morphology	Reorganization of the cytoskeletal structure	[164]
37	The human amniotic epithelial cells (FL)	0–50 μmol/L	PP2Ac, MMP−13	PP2A, PI3-K/AKT	Upregulation of PP2Ac mRNA and protein levels, opposing changes in PP2A	colorectal cancer	[165]
38	HepG2 cells	5–30 µM	Fas, FasL, NF-κB, p65	PP2A	Apoptosis reduces MC-LR-activated NF-κB, oxidative stress	Cell death	[166]
39	Human Embryonic Kidney cell line (HEK293)	-	β-catenin	p53, Akt, MAPK, JNK	Increased tumor-promoting activity and apoptosis in hepatocytes	Tumor	[167]
40	Wild-type HEK293 cells	50 nM	p53, PDK1, Akt, GSK−3β, β-catenin, c-myc, CDK2/cyclin E	p53, Akt, PP2A, MAPKs	Damage to DNA, trigger apoptosis, induction of cell cycle arrest	Cytotoxicity	[167]
41	CaCo2	0–200 µM	GST	-	Increased ROS level, affected enzymes activities	Cytotoxicity	[168]
42	Human embryonic kidney cells (HEK293)	0–50 nM	OATP1B1 or OATP1B3	PP2A A, MAPKs, ERK1/2, JNK, p38, MEK1/2	Apoptosis, increased ROS	Cytotoxicity	[169]
43	HepG2 cells	0–1 µg/mL	-	-	Intracellular ROS formation, DNA damage	Genotoxicity	[170]
44	Human colon carcinoma cell line (CaCo2), MCF−7	50 µM	LDH, intermembrane proteins	-	Decreased cell viability increased micro-calpain activity, ROS, and apoptosis.	Cytotoxicity	[171]
45	HepG2 cells	0.01, 0.1 and 1 µg/mL	Fpg, AraC and HU	PP1, PP2A	DNA damage, oxidative pyrimidine DNA damage, ROS production	Initiate tumor	[98]

**Abbreviations:** Bcl-2: B-cell leukemia/lymphoma 2 protein; cdc2: Cell division control 2; cdc25C: Cell division cycle 25; cGAS: Cyclic GMP-AMP synthase; cGAS-STING signaling pathway: Cyclic GMP–AMP synthase-stimulator of interferon gene signaling pathway; c-Myc: Cellular Myc; COX2: Cyclooxygenase enzyme; CTNNB1: Ctenin Beta 1; ERα: Estrogen receptors alpha; FAK: Focal adhesion kinase; FOXM1: Forkhead box M1; GS: Glycogen synthase; GSK-3: Glycogen synthase kinase 3; GSK3β: Glycogen synthase kinase 3 β; GSK3β: Glycogen synthase kinase 3 β; HAVSMCs: Human aortic vascular smooth muscle cells; HCC: Hepatocellular carcinoma; HIF-1α: Hypoxia-inducible factor 1-alpha; HL7702 cell: Human normal liver cells; HSP27: Heat shock protein 27; Hsp90: Heat shock protein 90; HUC-1 cells: Human uroepithelial cells-1 cells; HUVECs: Human umbilical vein endothelial cells; INS: Insulin; IRS1: Insulin receptor substrate 1; JAK2: Janus kinase 2; Krt16: Keratin 16; MAPK: Mitogen-activated protein kinases; MLC: Myosin light chain; MMP: Mitochondrial membrane potential; MMP7: Matrix metalloproteinase-7; mTOR: Mammalian target of rapamycin; P53: Tumor protein 53; PI3K: Phosphatidylinositol-3 kinase; PI3K: phosphoinositide 3-kinase; ROCK: Rho-associated protein kinase; ROS: Reactive oxygen species; S6K1: Ribosomal protein S6 kinase beta-1; SGC-7901 cells: Human gastric cancer cell line; STAT3: signal transducer and activator of transcription 3; VASP: vasodilator-stimulated phosphoprotein; VEGFCyclin D1: Vascular endothelial growth factor Cyclin D1; PPI3/AKT: phpsphoinositide 3-kinase/protein kinaseB; HL7702: Human normal liver cell line; OATP1B1: Organic anion transporting polypeptide 1B1; OATP1B3: Organic anion transporting polypeptide 1B3; L02: Human normal liver cell line; HepG2: Human hepatoma cell line; RWPE-1: Epithelial cell derived cell line; LX-2: Human hepatic stellate cell line; NCM460: Human normal intestinal epithelium cell line; HepG2: Human hepatoma cell line; L02: Human normal liver cell line: CaCo2: Cancer coli-2 cell line.

### 3.2 Impairment of mitochondrial nuclear crosstalk

While investigating the impact of MC-LR on mitochondrial dynamics, the mitochondrial nuclear crosstalk plays a crucial role [79]. It is widely recognized that mitochondria are MCs' most susceptible target organelles because the mitochondria regulate the electron transport chain (ETC) and oxidative phosphorylation (OXPHOS), which is notably comprised of five multi-protein complexes and are responsible for energy production [80]. Their biogenesis, structure, and cellular content highly influence these mitochondrial functions. In earlier studies, researchers recommended that the mitochondrial ETC and phosphorylation system were affected by MCs, which caused a decline in NADH dehydrogenase activity in rabbit liver. A major intracellular source of ROS is thought to be mitochondrial ETC, uncoupled by MC-LR, resulting in elevated ROS levels when the mitochondria become a vulnerable target of MC-LR [81]. It negatively impacts the mitochondrial electron transport chain and phosphorylation system, impairing NADH dehydrogenase activity, increasing ROS levels, and exacerbating oxidative stress. This highlights the need for further research on mitigating MC-LR's effects on mitochondrial function and ROS production. The production of ROS is one of the biochemical factors of MC toxicity [82]. The induction of ROS formation results in mitochondrial dysfunction. It is related to altered mitochondrial biogenesis, mitochondrial DNA (mtDNA) content, DNA strand breaks, and mutagenic oxidative DNA lesions through which MC-LR induces genotoxicity. Although mitochondria possess their genome, these organelles continuously communicate with the nucleus. The nuclear genome contains the genes for most of the mitochondrial proteins (1500–2000), so coordinated communication between the nuclear, cytoplasmic, and mitochondrial compartments is crucial [83]. Predominantly, two different signaling pathways affect mitochondrial structure/function and, consequently, cellular function, leading to mitochondrial diseases [84]. Anterograde signaling regulates oxidative phosphorylation (OXPHOS) and the biogenesis of mitochondria in exposure to environmental cues acquired by the nucleus. Retrograde signaling is a cellular response to mitochondrial respiratory malfunction and low mtDNA copy number, ultimately altering nuclear and mitochondrial gene expression and causing mitochondrial dysfunctions [85]. To permit transcription of nuclear-encoded mitochondrial genes that regulate oxidative phosphorylation, chromatin remodeling is triggered due to MCLR's influence on the change in crosstalk [86]. Pioneer transcription factors recruit the chromatin-modifying proteins via a co-activator. Similarly, the transcription associated with these genes and subsequent downstream mitochondrial observable traits, like mitochondrial DNA copy number, can be controlled by DNA methylation of regulatory sequences in nuclear-encoded mitochondrial genes, particularly DNA polymerase-γ (POLG) [87]. Furthermore, recent research indicates that nuclear regulators, such as DNA methyltransferases, transcription factors, and ten-eleven translocation (TET) demethylases, which are transferred from the nucleus to the mitochondria, could influence the transcription of the mitochondrial genome [88]. In contrast, MCLR also affects the nuclear epigenome retrograde signaling where mitochondrially-derived metabolites or secondary messengers, comprising S-adenosylmethionine (SAM), acetyl coenzyme A (acetyl Co-A), nicotinamide adenine dinucleotide (NAD^+^), ROS, flavin adenine dinucleotide (FAD), 2-oxoglutarate and calcium ions (Ca^2+^) affect DNA methylation and histone tail post-translational modifications, involving acetylation and methylation, in the nuclear epigenome as well as mitochondrial epigenome furthermore, it directly leads to the mtDNA depletion or heteroplasmy result from deleterious mtDNA mutations [89]. Mitochondrial genes are more susceptible to damage from ROS and other mutagenic factors because they lack histones and have a restricted mtDNA repair mechanism. Ultimately, mtDNA is left unrepaired and induces mtDNA damage [90]. MC-LR impairs mitochondrial biogenesis, oxidative phosphorylation, and nuclear regulation of mitochondrial genes through epigenetic modifications, contributing to mitochondrial diseases. Further research is needed to understand its effects on cellular and mitochondrial health fully.

### 3.3 Mitochondrial DNA damage

Mitochondria are essential for the tricarboxylic acid cycle (TCA), fatty encoding acid oxidation, calcium handling, controlling intrinsic apoptosis, and participating in the cell cycle. They possess their DNA, which encodes various proteins necessary for mitochondrial respiration [91]. Human cells have 16,569 base pairs of mtDNA, which encode 37 genes, including 13 polypeptides, two ribosomal RNAs, and 22 tRNAs [92]. DNA integrity is protected by a wide range of DNA damage-sensing signaling pathways and repair mechanisms in the nucleus [93]. These pathways usually compete to heal a lesion, preventing it from becoming stably mutated cells. Whereas only a few DNA repair pathways are active in mitochondria, and despite the common belief that this organelle lacks repair, mitochondrial base excision repair (mtBER) is quite robust. Despite being strong, mtBER cannot repair all mtDNA damage. The mtDNA is vulnerable to oxygen-free radicals and stress-induced injury as it lacks histones in the structure and efficient repair mechanisms. This increased response of mtDNA to oxidative lesions could either be due to the vicinity of the mitochondrial membrane region where ROS are produced or because of the asymmetric replication process of mtDNA. Furthermore, factors like the asymmetrical distribution of dNTPs in mitochondria promote higher levels of dGTP, may contribute to reduced fidelity during mtDNA replication, and increase the rate of spontaneous mutagenesis in the mitochondrial genome. mtDNA damage can cause mutation, trigger mitochondrial and cellular functions, commence a cascade of events that affect cell fate and apoptosis, and influence the transcription of mtDNA-encoded genes [94].

A change in mtDNA content could alter the expression of mitochondrial genes. MC-LR also targets ATP synthase, causing reduced ATP synthase activities that affect mitochondria's structure and function [95]. It can result in mitochondrial membrane rupture, ultrastructural disruption, and alterations to the MPT and MMP. MMP imbalance-induced mitochondrial dysfunction has a crucial function in the physiology of several ailments [96]. Mitochondrial dysfunctioning causes alterations in the biogenesis rate of mitochondria, mtDNA content, oxidative stress, and disturbed calcium homeostasis [97]. MC-LR-associated exposure causes mtDNA damage from endogenous and cytoplasmic changes induced by ROS, and mtDNA degradation may result from continuous mtDNA damage. Defective reparation or replication can cause mtDNA point mutations or omissions [98]. Controlling the fusion also improves mitochondrial respiratory function and enables genetic pairing by combining products with defective or depleted and intact mtDNA. Fission separates mitochondrial fragments with extremely damaged or weakened mtDNA. Mitophagy destroys the mitochondrial fragments due to mitochondrial membrane depolarization and increased ROS generation [99]. MtDNA destruction and mitophagy of faulty mitochondrial fragments can increase in response to elevated ROS or decreased DNA repair enzymes. Severe mtDNA damage results from the afflicted mitochondria exceeding the edge, which causes cellular malfunction and consequent end-organ damage to ensure severe mtDNA damage [100]. The p53 protein is activated by DNA damage primarily by DNA damage-responsive kinases by up-regulation of gene expression. Cell cycle arrest after DNA damage is brought on by the CDK inhibitor p21, the main downstream gene that activates p53 targets. More research revealed that MC-LR enhanced the levels of the genes p53, p21, and gadd45a, which are downstream regulators of p53 and oversee arresting the cell cycle and reconstruction of DNA, mdm2, a feedback regulator of p53 expression and activity, and the bax/bcl-2 ratio, which triggers apoptosis.

mtDNA mutations can arise 10–20 times more frequently than nuclear DNA (nDNA) [101]. This is why mutations in the mtDNA can lead to defective energy production, decreased ETC that produces ROS, and increased apoptosis. The phenotypic impacts of these mutagenic events in mtDNA are more severe than nDNA because all mutations in the mtDNA sequences occur in the coding sequences, raising the event's severity, and defective proteins are formed involved in mitochondria fission and fusion, leading to mitochondrial fragmentation [57]. Exposure to MC-LR significantly affects mitochondrial function and morphology by altering the transcription levels of mitochondrial genes, indicating broader disruptions in mitochondrial function and emphasizing the critical connection between mtDNA content and mitochondrial health. These changes highlight the potential for MC-LR to compromise mitochondrial stability, with implications for cellular health and disease progression, necessitating further investigation. ATP Synthase is also targeted by MC- LR, causing reduced ATP synthase activities. A decrease in ATP synthase activities would change how mitochondria look and work [95]. Disturbances inside mitochondria may result in sudden alterations of genetic and epigenetic mechanisms, resulting in disease development. The toxicity brought on by MC-LR is related to epigenetic alterations. Numerous ailments, including cancer, cardiovascular disease, neurological, age-related, and metabolism disorders, can be brought on by epigenetic alterations that result in mitochondrial malfunction [102].

## 4 Implication of MC-LR exposure on human health

Researchers found that when mice were given MC-LR dissolved in drinking water, it was observed that MC-LR in mouse brains damaged tight junctions and triggered abnormal immune and inflammatory activations. Microglia and astrocytes were activated by MC-LR exposure, which led to a neuroinflammatory response. It damages the blood-brain barrier (BBB) by stimulating the expression of matrix metalloproteinase-8 (MMP-8) regulated by NF-κβ, c-Fos, and c-Jun, posing a serious risk to human health [84]. This results in neuroinflammation due to the activation of microglia and astrocytes, highlighting a serious risk to brain health and underscoring the potential dangers of MC-LR to human neurological systems. Another report shows the development of benign prostatic hyperplasia (BPH) and prostate cancer by treatment with MC-LR. MC-LR could also induce the growth of microinvasion and prostatic intra-epithelial neoplasia (PIN). Colony formation, matrix metalloproteinase-2 (MMP-2), and matrix metalloproteinase-9 (MMP-9) secretion were elevated in normal human prostate epithelial (RWPE-1) cells that are characteristic of cancer cells. These MC-LR-altered prostate epithelial cells showed elevated expression of cyclooxygenase-2 (COX-2) and forkhead box M1 (FOXM1). Long-term exposure to MC-LR may also cause mouse brain abnormalities like Alzheimer's disease with neuronal cell death, decreased spine density, and an accumulation of Aβ and p-tau in the mouse brain. Following treatment to MC-LR, the mouse brain showed BBB impairment, alterations in dendritic morphology, reductions in dendritic spine density, and inflammatory responses, leading to cognitive impairments. MC-LR is toxic to both developmental and reproductive health. Proinflammatory cytokines and chemokines like TNF-α, MCP-1, IL-6, and CXCL10, PI3K/AKT/NF-κβ phosphorylation were shown to be upregulated in Sertoli cells, Leydig cells, and germ cells resulting in male reproductive toxicity known as orchitis showing symptoms such as spermatogenesis impairment and testicular inflammation. MC-LR causing female reproductive toxicity is shown to be more serious than male reproductive toxicity. In mice ovaries, MC-LR exposure induced ovarian cell apoptosis by activating ER stress and inhibiting PP2A activity, leading to follicular atresia, inflammation, apoptosis, and a decline in the gonadal index [103]. The risk of bladder cancer increases with higher levels of MC-LR as it permeates through bladder tissue, encouraging the proliferation and development of cancer in the bladder [104].

## 5 Analytical techniques for the determination of MC-LR

It is evident that continuous monitoring of the presence of MC-LR in water outputs is necessary to maintain the water's quality and protect public health. Numerous assays have already been invented to date for the estimation of MC-LR, like liquid chromatography-tandem mass spectrometry (LC-MS), enzyme-linked immunosorbent assay (ELISA), high-performance liquid chromatography (HPLC), colorimetric aptasensor assay (CAA), colorimetric inhibition assay (CIA), electrical immunoassay (EI), voltammetric aptasensor (VA), fluorescence biosensor immunoassay (FBI) [105]. Enzyme-linked immunosorbent assay (ELISA) is an excellent screening tool that can identify several MC variations and help show the presence of MCs releasing cyanobacteria. It rapidly detects MCs using a small amount of water. As the toxin can build up in seafood, ELISA can also identify MCs in various animal cells or tissues [106]. Biochemical methods include PPIA and ELISA. Due to the PP1 and PP2A inhibitory effects of MCs, PPIA can effectively detect MCs. PPIA is regarded as a convenient and inexpensive method of monitoring MCs. It is also quick and extremely sensitive to identify MCs and provides toxicological data to secure the health of humans and animals [107]. More studies have yet to be shown in Table 2. The HPLC approach needs the presence of cyano-toxin standards for accurate detection and quantification. While performing the analysis, sample preparation to concentrate the sample (MC-LR) is required. Solid-phase extraction (SPE) has been utilized in certain studies to reduce MC-LR and, subsequently, its HPLC-DAD (diode array detector) analysis. Nevertheless, these procedures require complex setup, significant heavy equipment, decent work skills, and a substantial time commitment, and their sensitivity and specificity could be better. Thus, due to their highly precise molecular identification and lack of time-consuming pre-treatment, biosensors are essential for both qualitative and quantitative detection of MC-LR. Despite its utility, improvements in sensitivity and specificity are needed to enhance the effectiveness of these procedures and streamLine the analysis of MC-LR. Future advancements should optimize these methods to achieve more reliable and efficient results.

**Table 2:** Table illustrates the detection of MC-LR from water samples using different analytical methods.

S. No.	Source	Analytical Methods	Sample	Amount of toxin detected	Inference	Reference
1	United States and China	ELISA	Surface water	-	Two matrix effect mitigation methods are examined to determine how China and the US pretreatment strategies affect the quantitative detection of cyanotoxins in surface water.	[172]
2	3 dams from Argentina	PPIA assay method	Algal bloom	0.4 µg/L	By MC spike recovery assay on a water sample, the PPIA method shows excellent correlation in MC-LR detection.	[1]
3	Huangpu River	ELISA and HPLC	*Microcystis aeruginosa* cultures in the laboratory and natural water samples	-	This study used HPLC and ELISA to identify the MC-LR content of Microcystis aeruginosa cultures in the lab and naturally occurring water samples from the Huangpu River in various seasons.	[173]
4	-	Fluorescent detection	water	-	Based on variations in fluorescence signal, 5-AF and 6-AF are small-molecule fluorescent probes useful for MC-LR detection in cells and water samples.	[174]
5	Water reservoirs located at the Ter River, in central Catalonia (NE Spain)	UHPLC-HRMS	Water sample	4–150 pg/L	This study describes developing a quick, sensitive, and reliable analytical technique for determining multiclass cyanotoxins. It is based on a dual SPE procedure using a polymeric cartridge, the Oasis HLB, and a graphitized non-porous carbon cartridge, the Supelclean™ ENVI-Carb™, followed by UHPLC-HRMS.	[175]
6	West Lake in the South China Agricultural University	FELISA	Water and fish sample	0.01–2.14 µg/L	Si-CDs were added to quantify MC-LR by changing their fluorescence intensity. HRP-catalyzed ABTS oxidation product (ox-ABTS) quenched Si-CD fluorescence.	[176]
7	Water	LC-MS/MS and ciELISA	Water sample	0.02–2.055 µg/L	The highly sensitive ciELISA detected nine MCs and NOD using MC-LR-keyhole limpet hemocyanin (KLH) for New Zealand white rabbit vaccination to produce antibodies.	[177]
8	Trampling Lake, Saskatchewan, Canada, and Pretzlaff Pond, Alberta, Canada	LC-HRMS/MS, 1 H and 13 C NMR spectroscopy, and UV spectroscopy	Canadian cyanobacterial bloom sample	-	The data show that [D-Leu1]-containing MCs may be more common in cyanobacterial blooms than previously thought yet are overlooked by concentrated LC-MS/MS screening.	[178]
9	Saline water of continental salt marsh in the Malahá (Granada, Spain).	UHPLC-DAD and UHPLC-MS/MS	SALLE	0.02–3.4 µg/L	UHPLC-MS/MS and UHPLC-DAD have been used in conjunction with a SALLE	[179]
10	Treated water of the Municipality of Macapá, Amazon River	LC-ESI-MS, ELISA	Water sample	0.026–2.1 µg/L	In this study, cyanotoxins and cyanobacteria were examined in samples taken from a drinking water treatment plant that uses the Amazon River. For the toxin analyses, ELISA, LC/MS, and molecular screening for cyanotoxin-producing gene candidates were used	[180]
11	Lake water from Dongwazi Lake (Nanjing, China), drinking bottled water from the supermarket	IC-ELISA-MscFv7-scFv	Water sample	0.471–0.548 µg/L	A mouse phage scFv library was successfully developed with a capacity of 8.67 107 CFU/mL was used to screen out 16 positive anti-MC-LR phage scFv particles in order IC-ELISA based on MC-LR	[181]
12	Water resources in Okinawa are in the subtropical region of Japan.	LC-MS and PP2A	-	-	Here, Microcystis bacteria expressing five MC variants were discovered and isolated using a dual assay (LC-MS analysis and PP2A inhibition assay)	[182]
13	Lake Uluabat	LC-MS/MS, LC-UV-MS, LC-HRMS and ELISA	Water column samples	0.2–330 µg/g	The lake samples included more than 36 MC variations, and a strain of M. aeruginosa (AQUAMEB−24) was recovered from Lake Uluabat using LC-MS/MS, LC-UV-MS, and a unique LC-HRMS technique.	[183]
14	Taihu Lake (Zhoutie town, Yixing, Jiangsu, China)	CE-ESI-MS	Water sample	0.2–1 µg/L	A study was done on the dynamic ranges and detection limits. Modifying the injection period, background electrolyte concentrations and pH values made the separation as effective as possible.	[184]
15	Antioquia, Colombia	UHPLC MS/MS	Water sample	-	For the first time, the UHPLC MS/MS analytical approach was used in this work to detect and quantify cyanotoxins in tropical reservoirs of northeastern Colombia. This allowed the toxins to be positively identified and validated. A technique was created and verified, showing that it was accurate, reproducible, and sensitive.	[185]
16	-	LFICA	Tap water	0.04 ng/mL	The molecular imprinting technique was combined with an enzyme-assisted colorimetric method to increase the sensitivity of conventional LFICA significantly. The findings indicated the neoteric LFICA method's enormous potential for sensitive MC-LR identification.	[186]
17	Vegetated lagoons (lagoon-B, lagoon-J, lagoon-S, and lagoon-D)	UHPLC-MS/MS	Water samples	1.301–11.630 ng/L	Using tandem mass spectrometry, UHPLC, and solid phase extraction, the constituents of MC-LR in four macrophyte-vegetated lagoons were identified. According to the findings, MC-LR was discovered in the lagoons of Nymphaea tetragona (lagoon-S), Vallisneria spiralis (lagoon-B), and another Vallisneria spiralis (lagoon-J).	[7]
18	Water sample	IC-ELISA-PAbs and scFv	-	0.44–1.36 µg/L	The anti-MC-LR phage scFv particles were screened using a rabbit phage display scFv library immunized with MC-LR, which had a 3.26 109 CFU/mL capacity.	[187]
19	Michigan Inland lakes	Adda-ELISA and LC-MS/MS	Water	0.6–3.8 ng/L	To quantify the 12 commercially available MCs and nodularin in surface and drinking waters, a high-throughput online concentration LC/MS/MS process has been created.	[188]
20	Lake Taihu (Jiangsu, China	MSPE (magnetic γ-CDP)-HPLC-MS/MS	Freshwater	0.8–2.0 pg /mL	A simple method for making the magnetic cyclodextrin polymer (Fe_3_O_4_@PDA@-CDP) composite was presented, and it was used as a novel adsorbent with superior capabilities for the MSPE of MCs.	[189]
21	Shallow water located on Poplar Island	Multi-hapten ELISA, Adda ELISA NMR spectroscopy, LC–MS 2 and LC-HRMS	Water	24.8–124 ng/g	Two broad-specificity MC ELISAs and LC-MS2 were utilized to evaluate free MCs, and MMPB and thiol de-conjugation procedures were employed to estimate 'total' MCs	[190]
22	Freshwater pond located in Changsha City	HPLC-ESI-MS	Water	-	The cyanobacterium's cyanotoxins were detected utilizing HPLC with an ultra-high resolution LTQ Orbitrap Velos Pro ETD mass spectrometry with electrospray ionization interface.	[191]
23	Porce II and Riogrande II water reservoirs located in the Antioquia, Colombia.	HPLC/MS, ELISA	Cyanobacterial bloom samples	124–5729 µg/L	HepG2 cells were used to analyze three crude algal blooms with MC-LR extracts for DNA damage using the comet assay. At 48 h, all extracts at 500 g mL−1 caused overall mortality, even at low concentrations, but only minor injury to hepatocytes exposed for 24 h.	[192]
24	-	Immunosensor	Water	0.034 µg/L	scFv has been used widely for immunodetection of MC-LR	[193]
25	Spain	Colorimetric protein phosphatase-inhibition assay	Water	0.05 μg/L	An MC-LR colorimetric protein phosphatase (PP) inhibition test exists. Molecular engineering created one recombinant, two natural, and one molecularly designed PP1 for this purpose.	[194]
26	Lake Erie	ELISA	Surface water	0.15–5 ppb	ELISA identifies MC-LR in surface water, suggesting that temperature, nutrient content, turbidity, wind speed, and direction affect Microcystis spp. density.	[195]
27	France	Colorimetric and fluorometric protein phosphatase inhibition method	drinking water	0.25 and 0.1 μg/L	A colorimetric and fluorometric protein phosphatase inhibition approach has been developed to directly detect MC-LR in drinking water without complicated clean-up stages and preconcentration procedures. It is cheaper, making it more attractive.	[196]

Abbreviations: HPLC-ESI-MS: High performance liquid chromatography/electrospray ionization tandem mass spectrometry; PCR: Polymerase chain reaction; HPLC: High-performance liquid chromatography; ELISA: Enzyme-linked immunoassay; NMR: Nuclear magnetic resonance; LC–MS: Liquid chromatography–mass spectrometry; LC-HRMS: Liquid chromatography-high-resolution mass spectrometry; MC: Microcystin; MMPB: 3-methoxy-2-methyl-4-phenylbutyric acid; HPLC/MS: High Performance Liquid Chromatography Mass Spectrometry; MC-LR: Microcystin leucine arginine; DNA: Deoxyribonucleic acid; ic-ELISA: indirect competitive enzyme-linked immunosorbent assay; PP2A:Protein Phosphatase 2A; UHPLC-DAD: Ultra high-performance liquid-chromatography-diode-array; UHPLC-MS/MS: Ultra high-performance liquid chromatography-tandem mass spectrometer; SALLE: Salting-out assisted liquid-liquid extraction; LC/MS/MS: Liquid chromatography tandem mass spectrometry; LC-UV-MS: Liquid chromatography with UV detection and mass spectrometry; LC-HRMS: Liquid chromatography-high resolution mass spectrometry; FELISA: Fluorescence enzyme- linked immunosorbent assay; LC-ESI-MS: Liquid Chromatography-Electrospray Ionization-Mass Spectrometry; Si-CDs: Silane-doped carbon dots; HRP: Horse radish peroxidase; UHPLC-HRMS: Ultra high performance Liquid Chromatography–High-Resolution Mass Spectrometry; UV-spectroscopy: Ultraviolet-visible spectroscopy; MSPE: Magnetic solid-phase extraction; Fe3O4@PDA-Au:Fe3O4 nanoclusters/polydopamine/gold nanoparticles; CE-ESI-MS: Capillary electrophoresis-electrospray ionization-mass spectrometry; PPIA: Peptidylprolyl isomerase A; LFICA: Lateral flow immunochromatographic assay; scFv: A single-chain fragment variable.

## 6 Sensing methods for MC-LR detection in water samples

MCs and associated toxins were identified and measured using water as a sample by various instruments, including ELISA and HPLC-MS. With the detection limit varying between 0.1 and 1 g/L, HPLC-MS is an analytical technique certified by the EPA in the United States for identifying different algal toxins [108]. However, when additional extremely abundant but less harmful homologs are present, it is challenging to discriminate between the structurally identical MC homologs. To assess low MC concentrations, a straightforward, user-friendly, quick, dependable, accurate, sensitive, and portable method may be utilized. The field of MC-LR environmental monitoring has progressed with the introduction of sensitive and precise biosensors. (Table 3).

**Table 3:** depicts the different advanced biosensors for detecting MC-LR in a water sample and the detection limit.

S. No.	Analytical method	Sensor	Sample	LoD value	Inference	Reference
1	Electrochemical impedance spectroscopy (EIS) **Advantage** - Utilizes advanced signal processing techniques and can investigate relaxations over a broad frequency spectrum. **Disadvantage -** It modifies the qualities particular to the system, such as the surface structure and sharpness.	Anti-MC-LR/MC-LR/cysteamine-coated SPCE	Natural water samples	0.69 ng L^−1^	An anti-MC-LR/MC-LR/cysteamine-coated SPCE biosensor was created to detect MC-LR in water. EIS was used to assess the sensor's performance.	[197]
		Microfluidic system	Drinking water	5.7 × 10^−10^ g L^−1^	It is the first documented system to distinguish between intracellular and extracellular MC-LR concentrations.	[198]
		AuNP-CNT	Water	2.8 ng/L	Dual-signal readout and enzyme-free immunoassay AuNP-CNt was developed to sensitively and accurately detect MC-LR.	[199]
2	Surface-enhanced Raman spectroscopy (SERS) **Advantage -** High sensitivity in detecting individual molecules, specific selectivity like fingerprints, and ability to work in water-based conditions. **Disadvantage** - Quantifying the quantity of ligand adsorbed may be challenging due to its unreliable nature.	AuNPs/graphene composite	Water	0.62 μM	The SERS-FET dual-mode biosensor, constructed using AuNPs/graphene composite as the sensing material, can increase the accuracy of test findings and provide more detection alternatives.	[200]
		SERS spectroscopic immunosensor	Freshwater	0.014 μg/L	SERS spectroscopic immunosensor with outstanding strength, selectivity, and sensitivity for detecting and measuring the MC-LR in aquatic environments was created.	[201]
		AuNPs	Tap water sample	0.002 ng/mL	Gold and magnetic nanoparticles were conjugated to the MC-LR aptamer and corresponding complementary DNA fragments. Then, cDNA-MNPs and MC-LR aptamer-AuNPs conjugates were utilized as signal and capture probes, respectively.	[202]
3	Photoelectrochemical (PEC) Advantages - They have a high sensitivity and can detect small biological compounds. Disadvantage - The operating procedure is intricate; the expense is considerable.	In_2_o_3_-in_2_s_3_-ti_3_c_2_ (io-is-tc) composite	Water sample	0.169 pmol/l and 17.4 pmol/l	This study used the IO-IS-TC composite to develop a potential dual-mode sensing platform with a photoanode matrix, greater output power, and obvious photocurrent response.	[203]
		Cus-tio_2_ nanocomposite	Lake water, river water, and tap water	2.0 × 10^5^ nm	This study detected aquatic MC-LR using a CuS-TiO2 heterojunction composite. TiO2 nanospheres received hydrothermally deposited CuS nanoparticles. To prevent electron-hole recombination and enhance photocurrent, the aptamer on the aptasensor trapped the photo-generated hole, which oxidized the analyte MC-LR.	[204]
		PEC immunoassay (CdS/TiO_2_ NRAs)	-	0.001 μg/L	CdS/TiO2 nanorod arrays (NRAs) immobilized visible-light-driven antigens in PEC immunoassay to detect MC-LR.	[205]
		Coo/Au/g-c3n4 nanocompsite	Lake water and tap water	0.01 pm	The PEC properties of a Coo/Au/g-C3N4 Z-scheme heterojunction were used to create a self-powered PEC aptasensor for MC-LR detection.	[206]
4	Aptasensor based biosensor. Advantages - Various immobilization and sensor creation methodologies for signal transduction, catalysis, and amplification. Disadvantages - biomolecule interactions cause rapid degradation in biological mediums.	CRISPR-Cas12a-based aptasensor platform	Freshwater	∼3 × 10^−6^ μg/L or 1 × 10^−3^ μg/L	A CRISPR-Cas12a-based aptasensor platform (dubbed MC-LR-Casor) is suggested for the on-site and sensitive detection of MC-LR.	[207]
		Aptasensor based biosensor		50 ng L^−1^	The TCM, an aptasensor based on the terahertz (THz) emission method, quickly and sensitively detects MC-LR.	[208]
		Aptamer@AuNps@Uio−66-nh_2_	Tap, pond, river water	0.004 ng/mL	Electrospinning, MOF seed generation, and AuNPs bridging aptamer procedures were employed to propose a new aptamer@AuNPs@UiO−66-NH2 electrospun nanofibrous coated fiber for microcystin-LR detection.	[209]
5	Fluorescent detection method Advantage - Quick reactions, non-invasiveness, great selectivity, and high sensitivity. Disadvantages - Enormous size, difficult to enhance by genetic modification and low photostability.	AuNPs	Tap and drinking water	0.83 nM	An aptamer protected by AuNPs and employed for sensitive MC-LR detection.	[210]
		Aptamer-based fluorescent sensor	Real water sample	138 pM	A fluorescence sensor for MC-LR selective identification was built using epoxy, SWNT immobilizers, DAP−10 aptamer, and unmodified Apt sensing ligand.	[211]
		Antibody-based optical planar waveguide	-	0.19 ng mL^−1^	The assay uses a cheap cartridge system with minimal reagent volumes and is highly repeatable.	[212]
		Phenyl-substituted diaza−18-crown−6 hydroxyquinoline (DCHQ-PH)	Drinking water	0.05 µg/L	An all-solid-state optical sensor for detecting MC-LR was prepared using phenyl-substituted diaza−18-crown−6 hydroxyquinoline (DCHQ-Ph).	[213]
		ICTS immunosensor	Water sample	0.1 μg/L	A novel immunosensor with quantum dots for microcystin-LR was created using ICTS.	[115]
		Wave guide system	Surface water sample	16 ± 3 ng/L	The immunosensor directly analyzed MCs in surface water samples, and the results matched those of standard LC-MS/MS.	[214]
6	Electrochemical techniques Advantages - Quick detection time, simple instrument operation, cheap cost, and portability. Disadvantage - Narrow or limited temperature range.	Electrochemical immunosensors with conductive nanobiochar paper	Environmental water	0.017 μg/L	Coating filter paper with highly conductive and dispersible nBC and anti-MCLR antibodies by dipping-drying created the paper immunosensor.	[215]
		ctDNA on gold electrode	Local water bodies	1.4 ng/L	Immobilizing ctDNA on gold electrodes creates biosensors. MC-LR alters immobilized ctDNA structure, decreasing electron transport resistance and boosting amperometric responsiveness.	[216]
		Graphene-gold nanocomposite	water	3.7 × 10 ^(−17)^ M	A graphene-gold nanocomposite method outperforms microcystin-LR sensors in sensitivity, repeatability, and stability.	[217]
		Graphene-based immunosensor	Environmental water sample	0.016 μg/L	A graphene-based immunosensor detects and amplifies microcystin-LR using horseradish peroxidase-carbon nanosphere-antibody.	[218]
7	Colorimetric method Advantage - High sensitivity, cost-effectiveness, ease of use, quick analysis, simplicity of operation, and clear visibility. Disadvantage - they cannot be reused, which prevents the ongoing usage of a single sensor.	Cu/Au/Pt TNs-encapsulated DNA hydrogel colorimetric biosensor	Tap water	3.0 ng L^−1^	For colorimetric MC-LR detection, Cu/Au/Pt TNs-encapsulated DNA hydrogel was created. This target-responsive and signal-amplification technology allowed the creation of colorimetric biosensors.	[219]
		(Fe₃O₄@SiO₂) and (PDA/CuNPs)	water samples	0.05 μg/L	Sandwich-type composites were magnetically separated by Fe3O4@SiO2PDA/CuNPs. The sensor worked with spiked samples, indicating visual and microcystin analog detection.	[220]
		Oligonucleotides AuNP dimers	-	-	For sensitive and selective MC-LR detection, the developed aptamer alters its structure to bind the target molecule and changes the solution color.	[221]
**Miscellaneous Biosensors**						
8	Phosphorescent immunosensor	MN-ZNS RTP QDS	Pond water	0.024 µg/L	Mn-ZnS RTP QDs recognize MC-LR with antigens and antibodies by binding correctly.	[30]
9	MINA	solid-phase polymerization on glass beads	Water	2.49 × 10–4 nmol L^−1^	MC-LR detection is done by colorimetric interaction between MC-LR and the enzyme HRP.	[222]
10	Competitive immunoassay	CNT@Co silicate nanocomposites and Fe3O4@PDA-AuNP	-	0.004 μg/L	The binding capability of biomolecules on nanomaterials and optimization of the conditions in the competitive immunoassay detected the MC-LR.	[223]
11	Double amplification	QCM	Water	100 ng/mL	QCM surface matrix was functionalized by monoclonal antibodies against MC-LR, and detection was achieved by primary amplification.	[224]
12	Resonant frequency change	PEMC	Water	dynamic range of 1 pg/mL to 100 ng/mL	Positive verification of MCLR detection was confirmed by a sandwich binding on the sensor with a second antibody binding to MCLR on the sensor.	[225]
13	SPR spectroscopy	SPR biosensor	Drinking water	73 ± 8 ng/L	The SPR biosensor can take four measurements in 60 minutes and utilize each SPR chip at least 40 times without losing binding capacity.	[214]
14	Raman spectroscopy, X-ray photoelectron spectroscopy, scanning electron microscopy, and a transmission electron micrograph.	SWNHs	Polluted water	0.03 μg/L	A sensitive electrochemical immunosensor that offers a biocompatible immobilization and sensitized recognition platform for analytes was proposed by functionalizing SWNHs with the analytes for MC-LR detection.	[226]
15	NMR	Magnetic nanoparticle	Lake water	0.6 ng/g	A sensitive and reliable toxin residue immunosensor was produced using magnetic nanoparticle relaxation.	[227]
16	ic-ELISA	Monoclonal antibody (Clone MC8C10) (Immunosensor)	Water	0.1 μg/L	Clone MC8C10 was produced and characterized with a high level of specificity against MC-LR, which ic-ELISA detects.	[228]

Abbreviations:

MB: Magnetic beads; Boron and nitrogen co-doped graphene quantum dots (BN-GQDS); Bismuth nanoparticles (BI NPs); SPR: surface plasmon resonance; ctDNA: calf thymus DNA; SWV: Square wave voltammetry; EIS: Electrochemical impedance spectroscopy; ICTH: Immunochromatographic test strips; CV: Cyclic voltammetry; AgNPs: Silver nanoparticles; MC-LR: microcystin-LR; QD: Quantum dot; PEMC: Piezoelectric-excited millimeter-sized cantilever; CNT@Co silicate: Carbon nanotube/cobalt silicate nanocomposites; Fe3O4@PDA-Au:Fe3O4 nanoclusters/polydopamine/gold nanoparticles; hpDNA-CuNCs: Hairpin DNA-templated copper nanoclusters; MINA: Molecularly Imprinted Nanoparticle-Based Assay, MINs: Molecularly Imprinted Nanoparticles;, QCM: Quartz crystal microbalance; AuNPs: Gold nanoparticles; AuNP-CNT: Enzyme-free immunosensor -gold nanoparticles-decorated-carbon nanotubes; SERS: Surface-enhanced Raman scattering spectroscopic; SERS-FET: Surface-enhanced Raman scattering -field effect transistor; FOCB: Fiber optical chemiluminescent biosensor; SWNHs: Single-walled carbon nanohorns; SWNTs: Single-walled carbon nanotubes; Fe₃O₄@SiO₂: Antibody-functionalized SiO₂-coated magnetic nanoparticles; ic-ELISA: indirect competitive enzyme-linked immunosorbent assay; NMR: Nuclear magnetic resonance; SWNHs: Single-walled carbon nanohorns; Cu/Au/Pt TNs: Cu/Au/Pt trimetallic nanoparticles; PEC: Photoelectrochemical; SPR: Surface plasmon resonance; LC/MS/MS: Liquid chromatography tandem mass spectrometry; CV: Cyclic voltammetry CV; SPCE: screen-printed carbon electrode; PDA/CuNPs: aptamer-functionalized polydopamine nanospheres decorated with Cu nanoparticles; NGH/Fe2O3: N-doped graphene hydrogel/hematite nanocomposites; PFC: Photofuel cell; APTES: 3-aminopropyl triethoxysilane; Clone MC8C10: Monoclonal antibody; DNA: Deoxyribonucleic acid; ELISA: Enzyme-linked immunoassay; HPLC: High-performance liquid chromatography; HRP: Horse radish peroxidase; LC-MS: Liquid chromatography–mass spectrometry; mAB: monoclonal antibody; MIPs: Molecularly imprinted polymers; nBC: nanobiochar particle; TCM: Terahertz chemical microscope; UV-spectroscopy: Ultraviolet-visible spectroscopy; CDP: Cyclodextrin polymer; MWCNT: Multiwalled carbon nanotubes.

### 6.1 Electrochemical sensors (ECS)

Electrochemical sensors are an excellent approach for detecting MC-LR since they may be automated, portable, and miniaturized for biosensing and are appropriate for in-situ observation. Electrochemical biosensors' detection signals are recorded as electron transfer between a specific dye and the electrode surface. For instance, the aptamer probe's attachment to MC-LR resulted in alterations in the structure and orientation of methylene blue (MB), which were then used by the sensor to transmit signals. The aptamer probe was deemed partially folded in the absence of MC-LR. The geometry of the aptamer probe altered when the aptamer and MC-LR were combined, which aided in the electron transport across the electrode interface and the MB. As a result, a greater current output was detected [109]. Another aptamer surface plasmon resonance (SPR) enhanced cathodic electrochemiluminescence sensor was designed using Bismuth nanomaterials (Bi NPs) as the SPR source and boron and nitrogen-doped graphene quantum dots (BN-GQDs) as the illuminator. The detection will be recorded when the fluorescent spectrum of BN-GQDs and the UV absorption spectra of Bi NPs coincide well. The SPR action of non-precious metal Bi NPs can trigger and increase the ECL signal of BN-GQDs [110]. An ECS composed of the gold electrode, which is physically immobilized by calf thymus DNA (ctDNA), reduces the electron transfer resistance of immobilized ctDNA in the presence of MC-LR with increased amperometric sensitivity. The sensor has a detection limit of 1.4 ng/L [111]. For highly potent MC-LR detection in water, an electrochemical aptamer-based sensor utilizing the redox facilitator hexaammineruthenium(III) chloride ([Ru(NH_3_)6]^3+^) was used. Edge of surface-confined [Ru (NH3)6]3+ separates in the presence of MC-LR, culminating in decreased faradaic potential from the reduction of [Ru(NH_3_)6]^3+^ to [Ru(NH_3_)6]^2+^ and a decline in square wave voltammetry. As an outcome, within 15 minutes of targeted incubation, SWV detected a significant signal reduction [112]. For MC-LR monitoring in Freshwater, an electrochemical aptatoxisensor was designed by immobilizing a thiolated aptamer on the surfaces of a dendrimeric GCE|SDD-Co(II) nanostructure. MC-LR selective affinity to the aptamer on GCE (SDD-Co(II)), the creation of a compound by AgNPs aptatoxisensor led to steric obstruction and electrostatic resistance, resulting in a modification of the maximum peak current of the electrochemical probe [113]. Optical sensor systems have a lower risk of producing explosives in a hazardous environment than electrical transmission systems and are thus clearer from electromagnetic disturbance. Since they can probe surface coatings utilizing a variety of optical phenomena while attaining low impedance and high sensitivity, optical sensors have a good deal of opportunity in this area. They also offer speed benefits, enable in-situ sensing, and enable real-time observations. It is also ideal for downsizing, distant, and multi-analyte detection.

### 6.2 Optical sensors

Optical biosensors have some clear advantages, including the absence of electrical connections and the ability to provide helpful results deprived of a power source. These can be enzymatic and non-enzymatic. In the case of non-enzymatic optical biosensors, the MC-LR detection occurs via charge distribution. MC-LR has two carboxylic acid groups (from DMe-Asp and D-Glu) and one guanidine group (from the L-Arg fragment). Depending on the associated pH, it may be found in distinct ionization forms in the solution under analysis. Considering the charge distribution of MC-LR in solution, a non-enzymatic sensor developed employing an optical Mg^2+^-selective sensor relies on diaza-18-crown-6 hydroxyquinoline (DCHQ-Ph), which is used for the indirect detection of MC-LR, monitoring changes in free cation ratios in response to exposure to various MC-LR contents could provide an indirect evaluation of the toxin [114]. For enzymatic detection, immunosensors are frequently employed. These sensors comprise fluorescent probes and labels. Sun et al. developed a similar immunosensor for MC-LR screening using immunochromatographic test strips (ICTS). The immunological sensor in ICTS used quantum dots as the fluorescent dye due to their good optical and electrical characteristics and extremely limited emission wavelength. The fluorescence probe level and MC-LR quantity impacted the ICTS's detectability [115]. Waveguide-based evanescent wave fluorescence biosensors have recently received much interest due to their ease of downsizing and sensitivity. Analytes percentage in samples may be determined by detecting and precisely relating fluorophore emission, which is stimulated by the excitation energy of the evanescent wave. Firstly, based on the evanescent wave principle, Shi et al. described an automated waveguide-based evanescent wave fluorescence immunoassay for the monitoring of MC-LR, where the evanescent wave transducer was a rectangle glass chip with a smooth 45° beveled solely on a single end face for light interaction [116]. Liu et al. have described a 32-analyte photonic fluorescence-based multi-channel waveguide biosensor incorporating fluidics, signal management, and analysis to detect MC-LR in the Lake water system. For analysis, the BSA-MC-LR complex was immobilized on the waveguide chip by (3-Mercaptopropyl) trimethoxysilane/N-(4-maleimidobutyryloxy) succinimide (MTS/GMBS), and it was demonstrated that it has homogeneous monolayer dispersion by atomic force microscopy. This sensor's applicability potential in assessing MC-LR in water samples was verified by the ability to identify all real lake samples, including those with MC-LR in the sub-microgram per liter range (for example, 0.5 g/L). Later, another waveguide-based fluorescence biosensor (TriPleXTM) was used for detecting environmental contaminants created by Liu et al. on a glass substrate employing the experimental analytes MC-LR, 2,4-D, atrazine, and BPA. For MC-LR, the TriPleXTM has a detection limit of 0.22 g/L [117], [118]. Optical biosensors based on QD-FRET are often employed in immunoassays, diagnostic tests, and assessments of biomolecular interaction. Moreover, the most advanced and popular detection probes for QD integration in bioanalyses are QDs antibody (Ab) conjugates. Feng et al. designed a hapten-coupled QD nanoprobe methodology for the fast and precise analysis of MC-LR in aqueous samples. Aminoethyl-MC-LR (H2N-etMC-LR), a compound responsible for the immunologic detection of the anti MC-LR antibody and photonic transmission, was conjugated with carboxyl QDs to create QD-hapten nanoprobes [119].

## 7 Strategies to protect against MC-LR-induced mitochondrial decay

Acute or chronic interaction with algal blooms is established as a risk factor for many chronic illnesses due to biological and chemical entities causing harm to the metabolic, hormonal, neurological, and immunological systems at low levels resulting from environmental exposure. In this context, the MC-LR received the most attention. Recent research indicates pathophysiological implications involving novel signaling pathways, including mitochondrial-nuclear crosstalk. The focus on mitochondrial function impairment and metabolic disturbance caused by MC-LR is not notable. The disruption of mitochondrial-nuclear crosstalk highlights significant concerns for cellular health and underscores the need for further investigation into these mechanisms. Since mitochondria are one of the principal generators of ROS, drugs that supply antioxidants to mitochondria, including vitamin C, vitamin E, creatine, or coenzyme Q, may be able to avert ROS overproduction. Medicines and dietary supplements that target mitochondria and drive cell death may be beneficial for protection [120]. An aberrant cellular proliferation, a hallmark of MC-LR-mediated hepatocellular carcinoma, can be controlled by specific mito-targeted therapies. Maintaining mitochondrial dynamics becomes easier with an improved diet that contains more antioxidants like polyunsaturated omega-3 fatty acids and flavonoids. Lipoic acid supplementation reduces oxidative stress by preventing lipid peroxidation [121]. A few distinct characteristics distinguish mitochondria and healthy and unhealthy mitochondria from other cellular compartments. These include the inner mitochondrial membrane's high membrane potential, the organelle's machinery for importing proteins, and the mitochondrial fusion process. These unique characteristics create tailored transport methods for physiologically active substances to and from mitochondria. A high membrane potential is negative within and acidic outside and is maintained via a transmembrane electrochemical gradient and pH difference. As a result, because of membrane potential, cation molecules are drawn to mitochondria and preferentially aggregate there. The drugs or chemicals are capable of being selectively directed into the mitochondria using a variety of strategies [122]. Among the techniques are ligands such as lipophilic cations, membrane fusion, mitochondria-penetrating peptides, mitochondrial protein import machinery, polymeric nanoparticles, liposomes, metal nanoparticles, polysomes, etc. Additionally, several mitochondrial-specific tools have been developed to safely transport physiologically active compounds to mitochondria, increasing the bioavailability of novel pharmaceutical formulations [123]. Research supports the idea that an imbalance of mitochondrial-nuclear crosstalk plays a crucial part in the genesis, advancement, and pathogenesis of several MC-LR-mediated human illnesses. Due to their function in the oxidation of substrates and ATP production, mitochondria are the primary location for the beginning and spread of metabolic disturbances. There are many unanswered questions even though exposure-related abnormalities in critical processes lead to decreased mitochondrial biogenesis, decreased mitochondrial content, mutations in mtDNA, increased oxidative stress, energy dysfunction, decreased antioxidant defense, and aberrant homeostasis of cytosolic calcium. Additionally, mitochondria cause inflammation and irreversible genomic and epigenetic alterations, which, together with other factors, contribute to developing degenerative diseases such as cancer [101]. Innovative methods that expand the degree to which mitochondrial defects contribute to developing these illnesses will be crucial for filling these information gaps. Selective targeting utilizing designed nano-vectors is an intriguing approach that could offer a new tool for mitochondria engineering and open the door to treating these catastrophic MC-LR-related mitochondrial illnesses in humans.

## 8 Mitigation strategies

Numerous strategies have been implemented to mitigate cyanobacterial blooms in aquatic environments. Physical and biological methods, including applying chemicals, algaecides, and phosphorus precipitation, have been utilized to diminish the severity of cyanobacterial blooms [124]. Physical control, involving the alteration of reservoir outflows to facilitate downstream flushing of cyanobacterial blooms, is a viable approach, particularly suitable for benthic cyanobacteria [125]. The efficacy of this method is influenced by factors such as proliferation size and physical catchment. However, this method is prohibitively expensive and pertinent only to vast water bodies. It is essential to consider the downstream receiving water bodies, particularly the aquatic invertebrate and fish ecosystems, at risk of severe impact [126]. The eradication of intracellular cyanotoxins (intact cells) can be achieved through various methods, including pre-treatment oxidation, coagulation/sedimentation/filtration, and membrane filtration [127]. One commonly used approach to neutralize HABs is pre-oxidation, which deactivates cyanobacterial cells. Pre-oxidizing agents such as potassium permanganate (KMnO_4_), chlorine (Cl_2_), and ozonation (O_3_) are frequently employed for this purpose [128]. Pre-chlorination is widely utilized to enhance the elimination of cyanobacterial cells. Upon penetration, chlorine interacts with cell membranes and intracellular materials, facilitating the release of algal organic matter (AOM) and toxins [129]. Consequently, the concentration of oxidizing agents must be optimized to dismantle dissolved toxins effectively. In their natural state, most cyanobacterial species, like microcystins, exist in colonial form [130]. During oxidation treatments, the fate of microcystins is confined to individual cells. Large, irregularly shaped microcystin colonies typically comprise thousands of cells per colony, posing challenges in determining their precise cell count through microscopy [131]. Flow cytometry is suitable for small sizes (0.1–50 μm) but not well-suited for large colonies. Consequently, treatment methods tailored for laboratory cultures comprising individual cells may not be suitable for cyanobacterial blooms. Advanced oxidation processes (AOPs) have garnered significant attention for their applications in drinking and wastewater treatment. These processes can degrade toxins, break down persistent organic compounds, and disinfect pathogens. AOPs generate and utilize the hydroxyl radical through both photochemical and non-photochemical means [132].

## 9 Conclusion and future directions

Cyanotoxin exposure is a major contributor to the disease development process. Our knowledge of the molecular pathways underlying the development of chronic ailments, including cancer, neurological disorders, cardiovascular disease, and respiratory disease, has been enhanced by recognizing the adverse effects of these pollutants on human cells. Here, we have discussed the mechanisms associated with MC-LR exposure-related signaling pathways and sensing methods for detecting MC-LR in the water sample. It is important to note that cyanobacteria can produce several cyanotoxins other than MC-LR, as typical algal blooms contain multiple cyanotoxins. Therefore, assessing how cyanotoxin combinations synergistically affect the human genome and epigenome is particularly interesting. It will be intriguing to unravel the precise effects of epigenetic alterations on gene expression, such as histone modification, DNA methylation, and non-coding RNAs. An emerging field of study is the molecular processes by which these toxins affect the epitranscriptome. Various analytical and sensing techniques could be more useful, including VA, EI, HPLC, LC-MS, CAA, ELISA, CIA, and FBI. More importantly, detecting toxin metabolites in the initial stages of harmful algal blooms before their actual release may help forecast episodes of infectious outbursts. The mitochondrial abnormalities in the etiology of several human illnesses linked to MC-LR exposure underline the translational relevance of innovative pharmacological treatments targeted at selective mitochondrial engineering, which might open new opportunities for developing novel therapies. The goal is to explain the risks of harmful cyanobacterial algal blooms and their evolution under environmental conditions leading to mitochondrial-induced epigenetic modifications. The most often found and researched cyanotoxin is microcystin. Additional research is underway to develop mitigation techniques for managing MC-LR pollution in drinking water. Strategies include using overly sensitive and specialized analytical methods to detect MC-LR and its metabolites in contaminated water. Various investigative and biochemical assays can be employed to estimate the contamination rate in water. One promising development in water quality monitoring is the emergence of optical biosensors that detect microcystins. These cutting-edge biosensors will change things up for detecting and preventing dangerous algal blooms. They use optics and biological identification aspects to their advantage. Improving and compressing these biosensors to be integrated into portable, field-deployable devices is one of the most promising future directions. Envision a world where portable optical biosensors could quickly and precisely determine whether local bodies of water have microcystin for environmental scientists and water treatment plant operators. These devices might monitor toxin levels in real-time, allowing quick reactions and actions to protect human health and the environment. There is tremendous promise in complementary optical biosensors with modern technologies like artificial intelligence and machine learning. These biosensors could detect microcystin and evaluate complicated patterns in water quality data using data analytics, allowing for incomparable precision in forecasting the emergence of algal blooms.

## Abbreviations

ADDA: Amino acids and aromatic 3-amino-9-methoxy- 2, 6,8-trimethyl-10- phenyldeca-4,6-dienoic acid
ATXs: Anatoxin-a
Bax: Bcl-2-associated X protein
BBB: Blood-brain barrier
Bcl-2: B-cell leukemia/lymphoma 2 protein
Bi NPs: Bismuth nanomaterials
BN-GQDs: Boron and nitrogen-doped graphene quantum dots
CAA: colorimetric aptasensor assay
CaMKII: Calcium/calmodulin-dependent protein kinase II
CIA: Colorimetric inhibition assay
CINC-2αβ: Cytokine-induced neutrophil chemoattractant-2αβ
COX-2: Cyclooxygenase-2
ctDNA: Calf thymus DNA
CXCL10: Chemokine interferon-γ inducible protein 10 kDa
CYNs: Cyclindrospermopsin
DNA-PK: DNA-dependent protein kinase
DO: Dissolved oxygen
DSB: Double-strand break
DSB-NHEJ: Double strand breaks-Non homologous end joining.
EI: Electrical immunoassay
ELISA: Enzyme-linked immunosorbent assay
ER: Endoplasmic reticulum
ETC: The electron transport chain
FBI: Fluorescence biosensor immunoassay
FOXM1: Forkhead box M1
GCLC: Glutamate-cysteine ligase catalytic
H2N-etMC-LR: Aminoethyl-MC-LR
HABs: Harmful algal blooms
HCC: Hepatocellular carcinoma
HPLC: high performance liquid chromatography
HPLC-DAD: High performance liquid chromatography -diode array detector
ICTS: Immunochromatographic test strips
IL-8: Interleukin-8
JNK: c -Jun N-terminal protein kinase
LC-MS: Liquid chromatography-tandem mass spectrometry
MAPK: Mitogen-activated protein kinases
MB: Methylene blue
MC: Microcystin
MC-LR: Microcystin-Leucine Arginine
MCP-1: Monocyte chemoattractant protein-1
MLC: Myosin light chain
MMP: Mitochondrial membrane potential
MMP-2: Matrix metalloproteinase-2
MMP-9: Matrix metalloproteinase-9
MPT: Membrane-permeabilization transition
mtDNA: Mitochondrial DNA
nAchR: Nicotinic acetylcholine receptor
NADPH: Nicotinamide adenine dinucleotide phosphate
nDNA: Nuclear DNA
NER: Nucleotide excision repair
NF-κβ: Nuclear factor kappa-light-chain-enhancer of activated B cells
NHEJ: Non-homologous end joining
NMs: Nanomaterials
NODs: Nodularins
NPs: Nanoparticles
OATP: Organic anion transporter peptides
OATP1B1: Organic anion transporting polypeptide 1B1
OATP1B3: Organic anion transporting polypeptide 1B3
OXPHOS: Oxidative phosphorylation
P53: Tumor protein 53
PI3K: Phosphatidylinositol-3 kinase
PIN: Prostatic intra-epithelial neoplasia
POPs: Pharmaceuticals and persistent organic pollutants
PP1: protein phosphatase-1
PP2A: Protein phosphatase 2 A
PPCPs: Personal care products
QD-FRET: Quantum Dot-Fluorescence resonance energy transfer
QDs: Quantum dots
ROS: Reactive oxygen species
SPE: Solid-phase extraction
SPR: Surface plasmon resonance
STXs: Saxitoxin
SWV: Square wave voltammetry
TNF-α: Tumor necrosis factor-alpha
VA: Voltammetric aptasensor

## CRediT authorship contribution statement

**Pooja Ratre:** Visualization, Validation, Software, Data curation. **Rupesh K. Srivastava:** Writing – review & editing. **Neelam Sahu:** Investigation, Formal analysis, Data curation. **Ram Kumar Nema:** Visualization, Data curation. **Pradyumna Kumar Mishra:** Writing – original draft, Validation, Supervision, Project administration, Formal analysis, Data curation, Conceptualization. **Nikita Soni:** Resources, Methodology, Investigation. **Kashish Gupta:** Writing – original draft.

## Declaration of Competing Interest

The authors declare that they have no known competing financial interests or personal relationships that could have appeared to influence the work reported in this paper.

## Data Availability

Data will be made available on request.
